# Nuclear and mtDNA phylogenetic analyses clarify the evolutionary history of two species of native Hawaiian bats and the taxonomy of Lasiurini (Mammalia: Chiroptera)

**DOI:** 10.1371/journal.pone.0186085

**Published:** 2017-10-11

**Authors:** Amy B. Baird, Janet K. Braun, Mark D. Engstrom, Ashlyn C. Holbert, Maritza G. Huerta, Burton K. Lim, Michael A. Mares, John C. Patton, John W. Bickham

**Affiliations:** 1 Department of Natural Sciences, University of Houston – Downtown, Houston, Texas, United States of America; 2 Sam Noble Museum, University of Oklahoma, Norman, Oklahoma, United States of America; 3 Department of Natural History, Royal Ontario Museum, Toronto, Ontario, Canada; 4 Department of Biology, University of Oklahoma, Norman, Oklahoma, United States of America; 5 Department of Forestry and Natural Resources, Purdue University, Lafayette, Indiana, United States of America; 6 Department of Wildlife and Fisheries Sciences, Texas A&M University, College Station, Texas, United States of America; University of Arkansas, UNITED STATES

## Abstract

Previous studies on genetics of hoary bats produced differing conclusions on the timing of their colonization of the Hawaiian Islands and whether or not North American (*Aeorestes cinereus*) and Hawaiian (*A*. *semotus*) hoary bats are distinct species. One study, using mtDNA COI and nuclear Rag2 and CMA1, concluded that hoary bats colonized the Hawaiian Islands no more than 10,000 years ago based on indications of population expansion at that time using Extended Bayesian Skyline Plots. The other study, using 3 mtDNA and 1 Y-chromosome locus, concluded that the Hawaiian Islands were colonized about 1 million years ago. To address the marked inconsistencies between those studies, we examined DNA sequences from 4 mitochondrial and 2 nuclear loci in lasiurine bats to investigate the timing of colonization of the Hawaiian Islands by hoary bats, test the hypothesis that Hawaiian and North American hoary bats belong to different species, and further investigate the generic level taxonomy within the tribe. Phylogenetic analysis and dating of the nodes of mtDNA haplotypes and of nuclear CMA1 alleles show that *A*. *semotus* invaded the Hawaiian Islands approximately 1.35 Ma and that multiple arrivals of *A*. *cinereus* occurred much more recently. Extended Bayesian Skyline plots show population expansion at about 20,000 years ago in the Hawaiian Islands, which we conclude does not represent the timing of colonization of the Hawaiian Islands given the high degree of genetic differentiation among *A*. *cinereus* and *A*. *semotus* (4.2% divergence at mtDNA Cytb) and the high degree of genetic diversity within *A*. *semotus*. Rather, population expansion 20,000 years ago could have resulted from colonization of additional islands, expansion after a bottleneck, or other factors. New genetic data also support the recognition of *A*. *semotus* and *A*. *cinereus* as distinct species, a finding consistent with previous morphological and behavioral studies. The phylogenetic analysis of CMA1 alleles shows the presence of 2 clades that are primarily associated with *A*. *semotus* mtDNA haplotypes, and are unique to the Hawaiian Islands. There is evidence for low levels of hybridization between *A*. *semotus* and *A*. *cinereus* on the Hawaiian Islands, but it is not extensive (<15% of individuals are of hybrid origin), and clearly each species is able to maintain its own genetic distinctiveness. Both mtDNA and nuclear DNA sequences show deep divergence between the 3 groups (genera) of lasiurine bats that correspond to the previously recognized morphological differences between them. We show that the Tribe Lasiurini contains the genera *Aeorestes* (hoary bats), *Lasiurus* (red bats), and *Dasypterus* (yellow bats).

## Introduction

Hoary bats (Lasiurini: *Aeorestes*) are unique among land mammals, in that they include the only extant mammal species native to the Hawaiian Islands. There are 4 recognized species in the genus *Aeorestes*: *A*. *semotus*, *A*. *cinereus*, *A*. *villosissimus*, and *A*. *egregius* [1]. *Aeorestes cinereus* occurs in North America and the Hawaiian Islands, *A*. *semotus* is restricted to the Hawaiian Islands, *A*. *villosissimus* is found in South America, and the more distantly related *A*. *egregius* occurs in Panama and northern South America, and previously was considered to be related to red bats based on morphology.

In 2015, 2 papers were published that addressed the colonization of the Hawaiian Islands by hoary bats [1,2] These studies used different approaches to estimate the number of colonizations of the Hawaiian Islands, and the approximate dates at which these occurred. Russell et al. [2] sequenced mitochondrial COI, and nuclear Rag2 and CMA1 (which they referred to as CHY; here we use the NCBI accepted abbreviation of CMA1 for the chymase gene). They utilized extended Bayesian skyline plots (EBSP) to understand historical population size changes in hoary bats and used them to estimate the time of colonization of Hawaii by lasiurines. They also reconstructed a maximum likelihood phylogeny and a maximum parsimony network for the COI locus alone. Their analysis included 59 hoary bats from the Hawaiian Islands, 85 hoary bats from North America, 2 South American hoary bats, and 1 sample each of *Dasypterus intermedius* and *D*. *xanthinus* as outgroups (although see Discussion below regarding sampling at each locus). Baird et al. [1] sequenced mitochondrial Cytb, ND1, ND2, and the Y-chromosomal DBY locus to conduct a molecular systematic revision of Lasiurini. They implemented maximum likelihood and Bayesian analyses of each gene separately, as well as *BEAST species tree analysis of combined data. They conducted a Bayesian dating analysis using BEAST to determine divergence times among clades (and therefore the first date of colonization of the Hawaiian Islands). They included 9 samples of Hawaiian hoary bats, 13 samples of North American hoary bats, 1 sample of South American hoary bats, and representative(s) of *A*. *egregius*, *Lasiurus atratus*, *L*. *blossevillii*, *L*. *borealis*, *L*. *pfeifferi*, *L*. *seminolus*, *L*. *frantzii*, *L*. *varius*, *D*. *ega*, *D*. *insularis*, *D*. *intermedius*, and *D*. *xanthinus*. Outgroups included *Eptesicus nilssoni*, *Myotis formosus*, *M*. *lucifugus*, *M*. *velifer* and/or *Tadarida brasiliensis*.

Russell et al. [2] and Baird et al. [1], despite the different methodologies and sampling schemes, found that the Hawaiian Islands were colonized multiple times by hoary bats, and that the geographic origin of the Hawaiian hoary bats was North America. They also found that there was at least 1 ancient colonization and multiple recent colonizations. However, their different approaches produced vastly different estimates of the timing of the ancient colonization. Russell et al. [2] concluded that the older colonization occurred no more than 10,000 years ago. Baird et al. [1] concluded that the ancient colonization occurred about 1 million years ago (0.4–1.8 Ma).

The main reason why the estimates of Hawaiian colonization differed by 2 orders of magnitude between Russell et al. [2] and Baird et al. [1] is the choice of methodologies. Russell et al. [2] employed the extended Bayesian skyline plot (EBSP), which was run separately on Hawaiian samples that group only with other Hawaiian samples (their “Hawaii1” clade; referred to as *A*. *semotus* in Baird et al. [1]) and Hawaiian samples that group with North American samples (their “Hawaii2” clade; referred to as *A*. *cinereus* in Baird et al. [1]). Russell et al. [2] used both mtDNA and nuclear DNA (Rag2 and CMA1) in the EBSP. Their results showed evidence for increased population size about 10,000 years ago for the “Hawaii1” (*A*. *semotus*) group. In the Discussion of the paper, they simply state that “EBSP analyses of this lineage support a model of population growth starting around 10 kya.” However, in the Abstract, they state (italic emphasis ours), “Coalescent demographic analyses of multilocus data suggest that modern populations of Hawaiian hoary bats were *founded no more than* 10 kya.” These 2 statements are quite different from one another. We agree that there is evidence of population expansion based on the EBSP; however, we do not agree with the conclusion that the population expansion represents the founding of hoary bat populations in Hawaii.

The approach taken by Baird et al. [1] relied on phylogenetic methods with the Hawaiian hoary bats in the context of their relationship to North American hoary bats and other lasiurine and vespertilionid relatives, rather than the population demographic-level EBSP of Hawaiian hoary bats in isolation taken by Russell et al. [2]. The phylogenetic approach utilized known fossil dates to calibrate the dates of the nodes on the phylogeny. Baird et al. [1] interpreted the date of the most recent common ancestor of the Hawaiian and North American hoary bat clades as the timing of diversification among the 2 lineages based on an analysis of mtDNA. Although it was not explicitly stated in the paper, Baird et al. [1] assumed the time at which the Hawaiian clade was isolated from the North American clade was both the time of divergence of the 2 taxa and the time of colonization of the Hawaiian Islands, since the morphological and genetic characters now associated with the *A*. *semotus* lineage have never been reported from hoary bats from the North American continent.

Given the degree of differentiation among the strictly Hawaiian lineage of hoary bats (*A*. *semotus*) and the North American/Hawaiian lineage (*A*. *cinereus*), we question the hypothesized divergence date estimate of 10,000 years proposed by Russell et al. [2]. In their study, they note about 3% sequence divergence among these lineages at COI. Baird et al. [1] reports about 4% divergence at Cytb. Traditionally, authors have cited an average rate of mtDNA divergence in mammals of 2% per million years, although Nabholz et al. [3] demonstrated that such generalization is inappropriate, as many taxa diverge at vastly different rates. Nabholz et al. [3] state that using a generic divergence rate of 2% per million years can over- or underestimate divergence times by a factor of 10. By that logic, even if the 4% divergence at Cytb were used to assume a divergence date, the minimum extreme expectation of divergence among the Hawaiian and North American hoary bats would be 200,000 years. That is still 20 times greater than the estimate of divergence derived in Russell et al. [2]. It is difficult to imagine how so much genetic change could have occurred among these 2 lineages in only 10,000 years (in addition to the morphological, behavioral, and acoustic differentiation among the groups). It is important to note that even with extensive sampling of North America (this study, [1,2]), no North American samples group with *A*. *semotus*; it is strictly limited to the Hawaiian Islands.

The Baird et al. [1] study was broader in scope than the phylogeographic study of Hawaiian hoary bats alone. It also examined phylogenetic relationships among most extant species of lasiurine bats and recommended taxonomic changes based on those findings. They proposed that the previously recognized subspecies of hoary bats should be elevated to species level. They also proposed that the red, yellow, and hoary bats should be placed in separate genera (*Lasiurus*, *Dasypterus*, and *Aeorestes*, respectively). Since the publication of Baird et al. [1], several authors have elected to use the taxonomic changes recommended therein (e.g., [4–7]).

One criticism of the taxonomic changes proposed by Baird et al. [1] was recently published. Ziegler et al. [8], in a paper describing a new genus and species of fossil bat from Hawaii, disagreed with the taxonomic revisions of hoary bats, and in an Appendix otherwise unrelated to the topic of their paper, disagreed with the division of Lasiurini into 3 genera. We outline and address their criticisms in the Discussion below.

In light of the 2 recent papers that produced conflicting conclusions to Baird et al. [1], we examined additional data from lasiurine bats for the following purposes: 1) to clarify the estimate of the timing of hoary bat colonization in the Hawaiian Islands and its relationship to the most recent common ancestor of the *A*. *semotus* lineage with a combined nuclear DNA and mtDNA analysis; 2) to determine the number of species of hoary bats that should be recognized by testing for the presence of gene flow between *A*. *cinereus* and *A*. *semotus* and testing for the monophyly of each lineage with both mtDNA and nuclear DNA; and 3) to present additional data to investigate the generic-level taxonomic changes to Lasiurini.

## Materials and methods

### Sampling

Our goal was to produce a dataset of combined loci from Baird et al. [1] and Russell et al. [2]. We aimed to have samples sequenced for genetic loci from both studies. Where available, sequences were obtained from GenBank. All new data generated by Russell et al. [2] (COI, CMA1, Rag2) and relevant sequences from Baird et al. [1] (ND1, ND2, Cytb) were included. Novel data produced in this paper include sequencing samples from Baird et al. [1] for COI, CMA1, and Rag2. Our final dataset contained 70 hoary bats from the Hawaiian Islands, 32 hoary bats from North America, 3 hoary bats from South America, and 1 representative sequence for each of the following lasiurine taxa for each gene: *L*. *blossevillii*, *L*. *borealis*, *L*. *pfeifferi*, *L*. *seminolus*, *L*. *frantzii*, *D*. *ega* from North America, *D*. *ega* from South America, *D*. *insularis*, *D*. *intermedius*, *D*. *xanthinus*, and *A*. *egregius*. Outgroups included *Myotis lucifugus* and *Tadarida brasiliensis*. DNA samples were not available for *L*. *atratus* and *L*. *varius* that were studied by Baird et al. [1] and are thus not included here.

### DNA amplification and sequencing

Available GenBank sequences were obtained for mitochondrial DNA genes cytochrome c oxidase I (COI), cytochrome-b (Cytb), NADH dehydrogenase I (ND1), and NADH dehydrogenase II (ND2), and nuclear recombination activating gene 2 (Rag2) and chymase (CMA1) genes. Primer names and amplification protocols follow those outlined in Baird et al. [1] (Cytb, ND1, ND2) and Russell et al. [2] (COI, Rag2, CMA1). Some species were difficult to sequence for CMA1 using the primers cited in Russell et al. [2], so we designed the following sequencing primers for CMA1: LAS CHY 801R seq (5’-AGGAGGAGGGAGGAGAGAGA) and LAS CHY 356F seq (5’- ACCATCCCTCTCAGTCTGCT). New sequences were deposited in GenBank and accession numbers can be found in Table 1. To sequence individual alleles for the nuclear loci, we designed allele-specific primers [9] when heterozygous individuals were encountered. A list of allele-specific primers used for each locus is found in Table 2. Sequences were aligned and edited using Geneious version 9.0.5 (http://www.geneious.com; [10]). Geneious was also used to calculate percent divergence values for Cytb sequences. These values are reported in Table 3.

**Table 1:** List of samples used and their associated GenBank accession numbers.

Species	Sample Number	Locality	mtDNA haplotype	CMA1 alleles	ND1	Cytb	ND2	COI	CMA1	RAG2	EBSP
A. cinereus^&^	AK11006	Queretaro	cinereus	cinereus	KT149023	KP341711	KT149103	MF990053	MF990094	MF990138, MF990139	
A. cinereus	AK11013	Queretaro	cinereus	cinereus	KT149068	KP341712	KT149112	MF990054	MF990095	MF990140, MF990141	
A. cinereus	AK11097	Queretaro	cinereus	cinereus	KT149062	KP341714	KT149106	MF990055	MF990096	MF990142, MF990143	
A. cinereus	AK11210	Queretaro	cinereus	cinereus		KP341715	MF990032	MF990056		MF990144, MF990145	
A. cinereus	AK11212	Queretaro	cinereus	cinereus	KT149063	KP341716	KT149114	MF990057	MF990097	MF990146, MF990147	
A. cinereus	ASK 1079	Texas	cinereus	cinereus	KT149064	KP341717	KT149110	MF990058	MF990098	MF990148	
A. cinereus	ASK 3520	Texas	cinereus	cinereus	KT149066	KP341718	KT149109	MF990059	MF990099, MF990100	MF990149	
A. cinereus	ASK4288*	Texas	cinereus					KR350014		KR350135, KR350136	
A. cinereus	ASK5155*	Arizona	cinereus					KR350015			
A. cinereus	BI56*	Canada: Alberta	cinereus					KR350016			
A. cinereus	BM126*	Tennessee	cinereus					KR350017			
A. cinereus	BM383*	Tennessee	cinereus					KR350018			
A. cinereus	BPBM185003	HI: Maui: Makawao	cinereus		KT149030	KP341721		MF990062		MF990151	2
A. cinereus	BPBM185519	HI: Maui	cinereus	cinereus			MF990037	MF990066	MF990106	MF990155	2
A. cinereus	BPBM185539	HI: Maui: Pukalani	cinereus	semotus	KT149058	KP341725	MF990039	MF990068	MF990108, MF990109	MF990157	2
A. cinereus	BPBM185541	HI: Maui: Olinda	cinereus	cinereus	KT149059	KP341726	MF990041	MF990070	MF990112		2
A. cinereus	CM82018*	West Virginia	cinereus					KR350019		KR350137, KR350138	
A. cinereus	FJB18*	HI: Maui	cinereus					KR350037			2
A. cinereus	FJB23*	HI: Maui	cinereus					KR350042			2
A. cinereus	FJB27*	HI: Maui	cinereus					KR350046		KR350091, KR350092	2
A. cinereus	FJB28*	HI: Maui	cinereus					KR350047			2
A. cinereus	FJB31*	HI: Maui	cinereus					KR350050			2
A. cinereus	FJB33*	HI: Oahu	cinereus					KR350052			2
A. cinereus	FJB60*	HI: Maui	cinereus	cinereus/semotus (F1)				KR350074	KR350009, KR350008	KR350125, KR350126	2
A. cinereus	FJB61*	HI: Maui	cinereus	semotus				KR350075	KR350010, KR350011	KR350127, KR350128	2
A. cinereus	FJB62*	HI: Maui	cinereus					KR350076		KR350129, KR350130	2
A. cinereus	FJB63*	HI: Oahu	cinereus					KR350077		KR350131, KR350132	2
A. cinereus	FJB64*	HI: Oahu	cinereus					KR350078		KR350133, KR350134	2
A. cinereus	JR07*	Michigan	cinereus					KR350079			
A. cinereus	LSUM350*	Michoacan	cinereus					KR350080			
A. cinereus	LSUM368*	Michoacan	cinereus					KR350081		KR350139, KR350140	
A. cinereus	MVZ199246*		cinereus					KR350082		KR350141, KR350142	
A. cinereus	NK 3562	New Mexico	cinereus	cinereus	KT149069	KP341728	KT149108	MF990071		MF990159	
A. cinereus	NK 3563	New Mexico	cinereus	cinereus	MF990020	KP341729	MF990042	MF990072		MF990160, MF990161	
A. cinereus	NK 3580	New Mexico	cinereus	cinereus	MF990021	KP341730	MF990043	MF990073	MF990113	MF990162	
A. cinereus	NK 3599	New Mexico	cinereus	cinereus	MF990022	KP341731	MF990044	MF990074	MF990114	MF990163	
A. cinereus	NK 3627	New Mexico	cinereus	cinereus	MF990023	KP341733	KT149111	MF990075	MF990115, MF990116	MF990164, MF990165	
A. cinereus	NK 3642	New Mexico	cinereus	cinereus	MF990024	KP341734	MF990045	MF990076	MF990117, MF990118	MF990166, MF990167	
A. cinereus	NK 6564	Sonora	cinereus	cinereus	MF990025	KP341735	MF990046	MF990077	MF990119, MF990120	MF990168, MF990169	
A. cinereus	NK 8096	Baja Cal.	cinereus	cinereus	KT149060	KP341736	KT149113	MF990078		MF990170, MF990171	
A. cinereus	NK 9191	New Mexico	cinereus	cinereus	MF990026	KP341737	MF990047	MF990079	MF990121	MF990172, MF990173	
A. cinereus	NK 9250	Sonora	cinereus	cinereus	KT149061	KP341738	KT149107	MF990080	MF990122	MF990174	
A. cinereus	NK 9273	Sonora	cinereus	cinereus	MF990027	KP341739	MF990048	MF990081	MF990123, MF990124	MF990175	
A. cinereus	RB5806*	Nebraska	cinereus					KR350083			
A. cinereus	RB5856*	Nebraska	cinereus					KR350084			
A. cinereus	RB5879*	Nebraska	cinereus					KR350085			
A. cinereus	SDBC04*	California	cinereus					KR350086			
A. egregius^&^	ROM17233 (F54554)	Guyana			KT149035	KP341745	KT149091	MF990087	MF990130	MF990182	
A. semotus	BPBM178452	HI: Hawaii: 5 mi N of Milolii turnoff	semotus		KT149028	KP341719	MF990033	MF990060		MF990150	
A. semotus	BPBM178453	HI: Hawaii: Volcano, Mauna Loa Estates	semotus		KT149029	KP341720		MF990061			
A. semotus^&^	BPBM185245	HI: Hawaii: Honaunau	semotus	semotus	KT149031	KP341722	MF990034	MF990063	MF990101	MF990152	1
A. semotus	BPBM185478	HI: Kauai: Kokee Road	semotus	semotus			MF990035	MF990064	MF990102, MF990103	MF990153	1
A. semotus	BPBM185479	HI: Maui: Haleakala National Park	semotus	semotus		KP341723	MF990036	MF990065	MF990104, MF990105	MF990154	1
A. semotus	BPBM185538	HI: Hawaii	semotus	cinereus	KT149065	KP341724	MF990038	MF990067	MF990107	MF990156	1
A. semotus	BPBM185540	HI: Hawaii: Hawi	semotus	semotus			MF990040	MF990069	MF990110, MF990111	MF990158	1
A. semotus	FJB01*	HI: Hawaii	semotus					KR350020			1
A. semotus	FJB02*	HI: Hawaii	semotus					KR350021			1
A. semotus	FJB03*	HI: Hawaii	semotus					KR350022			1
A. semotus	FJB04*	HI: Hawaii	semotus					KR350023			1
A. semotus	FJB05*	HI: Hawaii	semotus					KR350024			1
A. semotus	FJB06*	HI: Hawaii	semotus					KR350025			1
A. semotus	FJB07*	HI: Hawaii	semotus					KR350026			1
A. semotus	FJB08*	HI: Hawaii	semotus					KR350027			1
A. semotus	FJB09*	HI: Kauai	semotus					KR350028			1
A. semotus	FJB10*	HI: Hawaii	semotus					KR350029			1
A. semotus	FJB11*	HI: Hawaii	semotus					KR350030			1
A. semotus	FJB12*	HI: Hawaii	semotus					KR350031			1
A. semotus	FJB13*	HI: Hawaii	semotus					KR350032			1
A. semotus	FJB14*	HI: Hawaii	semotus					KR350033			1
A. semotus	FJB15*	HI: Hawaii	semotus					KR350034			1
A. semotus	FJB16*	HI: Hawaii	semotus					KR350035			1
A. semotus	FJB17*	HI: Hawaii	semotus					KR350036			1
A. semotus	FJB19*	HI: Maui	semotus					KR350038			1
A. semotus	FJB20*	HI: Hawaii	semotus					KR350039			1
A. semotus	FJB21*	HI: Hawaii	semotus					KR350040			1
A. semotus	FJB22*	HI: Hawaii	semotus					KR350041			1
A. semotus	FJB24*	HI: Kauai	semotus					KR350043			1
A. semotus	FJB25*	HI: Hawaii	semotus					KR350044			1
A. semotus	FJB26*	HI: Hawaii	semotus					KR350045			1
A. semotus	FJB29*	HI: Hawaii	semotus					KR350048			1
A. semotus	FJB30*	HI: Hawaii	semotus					KR350049			1
A. semotus	FJB32*	HI: Hawaii	semotus					KR350051			1
A. semotus	FJB34*	HI: Oahu	semotus					KR350053			1
A. semotus	FJB35*	HI: Hawaii	semotus					KR350054		KR350093, KR350094	1
A. semotus	FJB36*	HI: Hawaii	semotus	semotus				KR350055	KR349974, KR349974		1
A. semotus	FJB37*	HI: Hawaii	semotus	semotus				KR350056	KR349977, KR349976	KR350095, KR350096	1
A. semotus	FJB38*	HI: Hawaii	semotus	semotus				KR350057	KR349978, KR349979		1
A. semotus	FJB39*	HI: Hawaii	semotus	semotus				KR350058	KR349980, KR349981		1
A. semotus	FJB40*	HI: Hawaii	semotus	semotus				KR350059	KR349982, KR349983		1
A. semotus	FJB41*	HI: Hawaii	semotus	semotus				KR350060	KR349984, KR349985	KR350097, KR350098	1
A. semotus	FJB42*	HI: Hawaii	semotus	semotus				KR350061	KR349986, KR349987	KR350099, KR350010	1
A. semotus	FJB43*	HI: Hawaii								KR350101, KR350102	
A. semotus	FJB44*	HI: Hawaii	semotus	semotus				KR350062	KR349989, KR349988	KR350103, KR350104	1
A. semotus	FJB45*	HI: Hawaii	semotus					KR350063		KR350105, KR350106	1
A. semotus	FJB46*	HI: Hawaii	semotus	semotus				KR350064	KR349991, KR349990	KR350107, KR350108	1
A. semotus	FJB47*	HI: Hawaii	semotus					KR350065		KR350109, KR350110	1
A. semotus	FJB49*	HI: Hawaii	semotus	semotus				KR350066	KR349992, KR349993	KR350111, KR350112	1
A. semotus	FJB52*	HI: Hawaii	semotus	semotus				KR350067	KR349995, KR349994	KR350113, KR350114	1
A. semotus	FJB53*	HI: Hawaii	semotus	semotus				KR350068	KR349997, KR349996	KR350115, KR350116	1
A. semotus	FJB55*	HI: Hawaii	semotus	semotus				KR350069	KR349998, KR349999	KR350117, KR350118	1
A. semotus	FJB56*	HI: Hawaii	semotus	semotus				KR350070	KR530000, KR350001		1
A. semotus	FJB57*	HI: Hawaii	semotus	semotus				KR350071	KR350002, KR350003	KR350119, KR350120	1
A. semotus	FJB58*	HI: Hawaii	semotus	semotus				KR350072	KR350004, KR350005	KR350121, KR350122	1
A. semotus	FJB59*	HI: Hawaii	semotus	semotus				KR350073	KR350006, KR350007	KR350123, KR350124	1
A. villosissimus^&^	NK11502	Bolivia			KT149033	KP341727	KT149081	MF990082		MF990176	
A. villosissimus	M260258*	Bolivia						KR350012			
A. villosissimus	M268079*	Galapagos Is.						KR350013			
D. ega^&^	NK15304	Bolivia			KT149045	KP341743	MF990051	MF990086	MF990128, MF990129	MF990181	
D. ega^&^	AK7693	Belize			KT149040	KP341741	MF990050	MF990085	MF990127	MF990179, MF990180	
D. insularis^&^	TK32049	Cuba			KT149053	KP341747	KT149098	MF990088	MF990131, MF990132	MF990183, MF990184	
D. intermedius^&^	AK11148 or ASK0422	Mexico			KT149048	KP341748	KT149100	MF990089	MF990133	MF990185	
D. xanthinus^&^	NK11103	New Mexico			KT149056	KP341757	KT149093	MF990093	MF990137	MF990189	
L. blossevillii^&^	ROM111049, ROM111055, or AK13464				KT148900	KP341705	MF990049	MF990083	MF990125	MF990177	
L. borealis^&^	AK7214 or AK21072	Illinois or Kansas			KT148978	MF990028	KT149076	MF990084	MF990126	MF990178	
L. frantzii^&^	AK11119	Tamaulipas			KT149016	MF990031	MF990052	MF990092	MF990136	MF990188	
L. pfeifferi^&^	TK32056	Cuba			KT149006	MF990029	KT149084	MF990090	MF990134	MF990186	
L. seminolus^&^	AK10354 or AK1565	Texas			KT149011	MF990030	KT149085	MF990091	MF990135	MF990187	

An asterisk next to sample name indicates that data was generated in Russell et al. [2]. The column “mtDNA haplotype” shows what species the mtDNA groups with (for hoary bats only). The column “CMA1 nuclear alleles” indicates what species the CMA1 alleles group with (for hoary bats only). Cells highlighted in orange show samples for which there is a mismatch between the mtDNA haplotype and CMA1 allele(s) indicating potential hybrid ancestry. Cells with two accession numbers for the nuclear loci indicate that two alleles were sequenced for that locus (heterozygous). The column “EBSP” indicates which scenario of EBSP the sample was used in (see text). Samples included in the dating analysis are indicated by a “&” superscript in the first column. HI = Hawaii. Specific localities for Hawaiian samples are given where available (these data were not provided for samples completed by Russell et al. [2]).

**Table 2:** Allele-specific primers used in nuclear gene sequencing.

Primer Name	Primer sequence	Locus
LAS CHY 429FA	GGGATAACAAGAAGGAAAAGAAAAAGA	CHY
LAS CHY 429FG	GGGATAACAAGAAGGAAAAGAAAAAGG	CHY
LASCHY785RA	AGAGAGAGAGGGGTGGGA	CHY
LASCHY785RG	AGAGAGAGAGGGGTGGGG	CHY
LASCHY739RG	CAGGAAAGTCATCTACTGCTACCCAG	CHY
LASCHY739RT	CAGGAAAGTCATCTACTGCTACCCAT	CHY
LAS RAG 194FG	AAGATGTATGTGATGTCTGTGG	Rag2
LAS RAG 194FT	AAGATGTATGTGATGTCTGTGT	Rag2
LAS RAG 250FC	CACTGAGAAAGACTTGGTAGGC	Rag2
LAS RAG 250FA	CACTGAGAAAGACTTGGTAGGA	Rag2
LAS RAG 250FT	CACTGAGAAAGACTTGGTAGGT	Rag2
LAS RAG 250RG	ATCTGGCTTCAGGGACATCG	Rag2
LAS RAG 250RT	ATCTGGCTTCAGGGACATCT	Rag2
LAS RAG 124FA	GGCTTAGAGTCGGAAAGGCAA	Rag2
LAS RAG 124FG	GGCTTAGAGTCGGAAAGGCAG	Rag2
LAS RAG 761RA	TCCAATCTGGGGTCTCCATCTCA	Rag2
LAS RAG 761RT	TCCAATCTGGGGTCTCCATCTCT	Rag2

The last letter of the primer name indicates the base to which the primer is specific. The second to last letter of the primer name indicates whether the primer is a forward (F) or reverse (R) sequencing primer. All sequences are read in the 5’ to 3’ direction.

**Table 3:** Percent sequence divergence values for Cytb.

	1	2	3	4	5	6	7	8	9	10	11	12	13	14
*A*. *cinereus* (1)	0.65													
*A*. *semotus* (2)	4.23	0.27												
*A*. *villosissimus* (3)	10.38	10.15												
*A*. *egregius* (4)	16.68	16.90	16.14											
*L*. *blossevillii* (5)	19.54	20.04	17.54	19.21										
*L*. *borealis* (6)	18.33	18.82	17.44	17.62	15.21									
*L*. *pfeifferi* (7)	18.99	19.37	18.33	17.28	13.72	8.38								
*L*. *seminolus* (8)	18.34	18.62	18.53	19.07	14.95	9.13	5.03							
*L*. *frantzii* (9)	21.34	21.15	18.98	20.65	12.72	14.81	14.71	15.12						
*D*. *ega NA* (10)	19.83	19.69	19.91	17.72	20.35	19.23	19.27	19.34	21.27					
*D*. *ega SA* (11)	19.71	18.68	19.65	18.86	20.70	19.41	19.27	19.34	22.00	8.95				
*D*. *insularis* (12)	19.59	19.07	19.04	18.68	19.56	19.77	19.16	19.96	19.71	13.95	15.26			
*D*. *intermedius* (13)	18.85	18.59	19.39	17.46	20.09	20.04	18.95	19.43	20.54	13.95	12.90	11.40		
*D*. *xanthinus* (14)	17.73	17.48	17.98	17.54	19.04	18.87	18.85	19.52	21.06	15.44	16.49	15.09	15.35	

Numbers along diagonals represent within-species diversity if >1 sample was sequenced for that species. Numbers below diagonals represent between-species divergence. Values are represented as percentages. NA = North America; SA = South America.

### Phylogenetic analyses

Sequence data from each locus were initially analyzed independently. The program jModelTest version 2.1.10 [11–12] was used to obtain the appropriate model of evolution for each locus. The model was implemented in a Bayesian phylogenetic analysis using MrBayes version 3.2 [13–14]. Each Bayesian tree was run for 5 million generations with a sample frequency of 1,000 generations. Tracer version 1.6 [15] was used to determine that runs had reached stationarity and that a 25% burnin was appropriate. All trees were visualized using FigTree version 1.4.0 [16].

### Haplotype/Allele networks

Network relationships for the hoary bats at the CMA1 and COI loci were conducted independently using the TCS network setting [17] in PopArt version 1.7 (http://popart.otago.ac.nz). Note that not all individuals sequenced for mtDNA were sequenced for CMA1, including a majority of the samples from Russell et al. [2]. Rag2 was not used in further analyses due to its inability to resolve *A*. *semotus* and *A*. *cinereus*, along with other well-established relationships of lasiurine species. See the Results for further explanation.

Each allele sequenced was used in the CMA1 network analysis; therefore, each individual is represented by 2 alleles in the network. Two different networks for CMA1 were produced. The first included all North American and Hawaiian samples for which the sample’s corresponding mtDNA haplotype group was known. The purpose of the first network was to visualize individuals of hybrid ancestry as having mismatches of mtDNA haplotype and CMA1 nuclear alleles. The second CMA1 network included only Hawaiian samples to examine the geographic distribution of *A*. *cinereus* vs. *A*. *semotus* alleles on the Hawaiian archipelago. Available CMA1 sequences came from individuals from the islands of Hawaii, Maui, and Kauai.

For the COI networks, a similar approach was taken. The first COI network included all North American and Hawaiian samples for which the CMA1 allele group was known. Again, this served to visualize mismatches of mtDNA haplotype and CMA1 nuclear alleles within individuals. The second network included all samples sequenced for COI and was colored based on the geographic locality of each sample. Samples available in the second network included individuals from North America, and the islands of Hawaii, Maui, Kauai, and Oahu.

### Historical demography

Historical population demography was explored using Extended Bayesian Skyline Plots (EBSP). These were implemented in the program BEAST v. 2.4.4 [18] with a combination of COI and CMA1 sequence data [19]. Other mitochondrial loci were eliminated based on significantly lower sample sizes for those loci. Two different scenarios were tested (species are defined based on their mitochondrial haplotypes): 1) only *A*. *semotus*; 2) only *A*. *cinereus* on the Hawaiian Islands. jModelTest v. 2.1.10 was used to determine the appropriate model of evolution for each locus under each scenario. To maintain consistency with the EBSP analysis conducted by Russell et al. [2], we specified many of the same parameters for analysis. The mtDNA substitution rate was specified as 2% per million years. The CMA1 clock rate prior was set as uniform with an upper value of 1. Operator values were set as specified by Russell et al. [2]: kappa values were given a weight of 2 and a weight of 15 was given for substitution rates and heights. *N*_*e*_ (effective population size) was calculated assuming an average generation time of 2 years, as in Russell et al. [2]. Some parameters were not specified in Russell et al. [2] but were set to the following in our analyses: both clock models were set to strict. COI was set as the reference sequence for the clock rate (2% per million years) and CMA1 was estimated. Tree models were set to coalescent extended Bayesian skyline and the population factor for the CMA1 tree model was set to 2 (diploid) and for COI to 0.5 (haploid, maternally inherited). Each scenario was run until convergence was reached (as determined by visualizing the trace logs in Tracer v. 1.6).

### Divergence dating

Divergence time estimates were generated using the program BEAST 2.4.4 [18] based on sequences from Cytb, COI, ND1, ND2 and CMA1 for 1 representative of each species. Representative samples for each species were chosen based on the completeness of their gene sequences. The *A*. *semotus* representative (BPBM 185245) was chosen because it is the only sample not of hybrid origin for which all genes were successfully sequenced. The representative *A*. *cinereus* (AK11006) was chosen because it had complete sequence for all genes. We wanted a representative from North America, rather than a Hawaiian *A*. *cinereus* because the goal of this analysis was to date the divergence between North American and Hawaiian *A*. *semotus*. The distance between these two samples can be seen in each of the gene trees (Supporting Information). *Myotis lucifugus* was used as an outgroup in addition to other members of Lasiurini. From some outgroup taxa, no single individual was sequenced for all genes, so sequences from different individuals were used to represent these taxa (hoary bat sequences were each derived from only a single individual). Table 1 indicates the sequences that were used in the divergence analysis. The program jModelTest was used to find appropriate models of evolution for each gene (using 3 substitution schemes) for this reduced sample set.

For the BEAST analysis, trees and clocks were linked across loci and sites were unlinked. The clock model was set to relaxed log-normal and the tree prior was set to birth-death. To calibrate the nodes, fossil date estimates from *Khonsunycteris* (>34 Ma) [20] and *Lasiurus* (>11.6 Ma) [21] were specified as minimum ages for the nodes representing the most recent common ancestor of *Myotis*/Lasiurini, and the common ancestor of Lasiurini, respectively. As precedent for how to establish these priors, we followed Amador et al. [4] in their use of these fossils to calibrate nodes within their larger Chiropteran phylogeny. BEAST settings for M, S, and Offset regarding these fossils are the same as those specified in Table 3 of Amador et al. [4]. A log-normal distribution prior for node ages was applied. The fossil-calibrated nodes were defined as monophyletic.

## Results

### Sequence success

**Fig 1:**
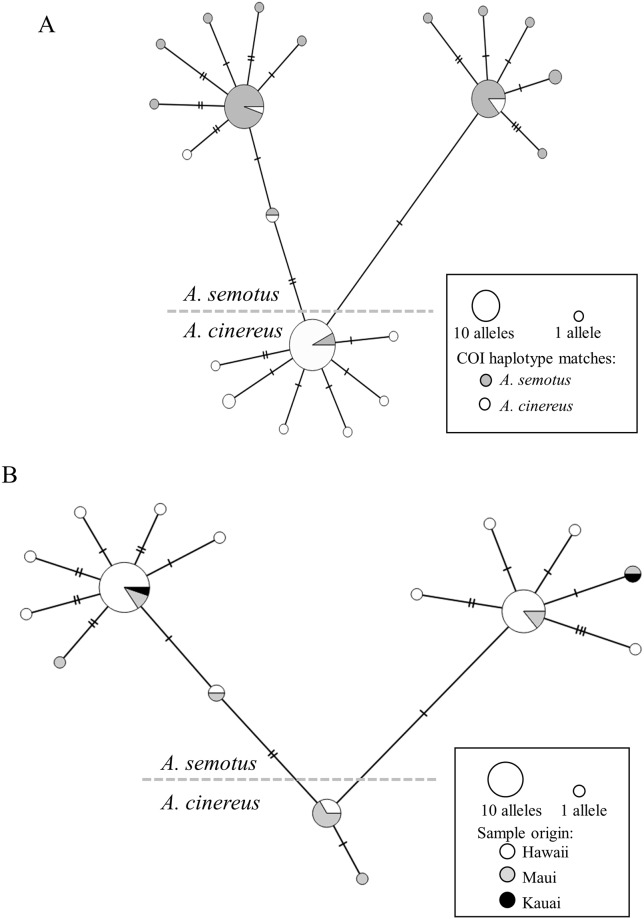
Allele networks based on nuclear CMA1 sequences. All alleles were used in the network; therefore, the total number of individuals is ½ the total number of alleles. A) Samples from North America and Hawaii from which sequences are available for both CMA1 and mtDNA. The network is based only on CMA1 sequence, but is colored to show how the sample’s corresponding mtDNA sequences group (either with *A*. *semotus* or *A*. *cinereus*). B) All samples from the Hawaiian Islands, colored by where the sample was collected. Any differences in the networks for parts A and B are due to the North American samples not being included in part B, and all Hawaiian samples (not just those also sequenced for mtDNA) were included in part B.

All samples successfully sequenced are those with GenBank numbers in Table 1. All samples sequenced for CMA1 had 2 alleles sequenced, so heterozygotes have 2 GenBank numbers per sample. For Rag2, we were unable to resolve the alleles for some heterozygous individuals. For those individuals, the consensus sequence of the 2 alleles was used in the Rag2 gene tree. Table 3 depicts the within- and among-species sequence divergence for the Cytb gene. In CMA1, a surprisingly high level of diversity was observed within *A*. *semotus*, greater than the level of diversity within the more broadly geographically distributed *A*. *cinereus* (Fig 1 and S5 Fig). We explored explanations for this high diversity of CMA1 by testing for selection in CMA1 in *A*. *semotus*. Those tests were negative (data not shown). Additionally, an insertion of 222–228 base pairs was present in some yellow bat species in CMA1. It was present in *D*. *ega* (North and South American forms), *D*. *insularis*, and *D*. *intermedius* but not *D*. *xanthinus*. Therefore, the insertion likely arose after *D*. *xanthinus* split with the common ancestor of the remaining yellow bats. The insertion was not included in the phylogenetic analysis of CMA1.

### Gene trees

Models of evolution implemented for gene tree reconstruction included TPM2uf+I+G for Cytb, TIM+I+G for ND1, TIM2+I+G for ND2, TrN+I+G for COI, TIM3ef+G for CMA1, and HKY+I+G for Rag2. Individual gene trees for mitochondrial COI, Cytb, ND1, and ND2 and nuclear CMA1 and Rag2 are shown in S1–S6 Figs. The nuclear gene trees are depicted with individual alleles as tips on the trees, where available. Different alleles from the same individual are labelled with the sample name followed by “A” or “B” as a suffix. Homozygous samples are labelled with “AA” as a suffix. Unresolved sequences (in the Rag2 gene tree) are labelled with “UR” as a suffix. This indicates that a consensus sequence of the two alleles was used in the phylogenetic analysis.

Gene trees from Cytb, ND1, and ND2 generally agree with the corresponding trees presented in Baird et al. [1]. All mitochondrial gene trees show clear evidence of distinct clades for *A*. *semotus* (restricted to the Hawaiian Islands) and *A*. *cinereus* (samples from North America, Maui, and Oahu). This study and Baird et al. [1] differed by their use of different outgroup taxa and different numbers of individuals that were sequenced. The one inconsistency between the results of our study and Baird et al. [1] lies in the ND2 gene tree. The red and hoary bats are shown as sister taxa at the ND2 locus, with the yellow bats sister to those two; Baird et al. [1] showed the hoary bats and yellow bats as sister taxa at the ND2 locus, though that relationship was weakly supported. In the present study, all gene trees agree that the red and hoary bats are sister taxa and the yellow bats are more distantly related, with the exception of COI which shows the red and yellow bats as sister taxa. Note that at a Bayesian posterior probability of 0.78, the relationship of red and yellow bats as sister taxa in COI is the lowest level of support among the three putative genera across all of the gene trees. Additionally, all mtDNA gene trees show strong support for *A*. *egregius* sharing a common ancestor most recently with the remaining hoary bat (*Aeorestes*) species.

The CMA1 locus appeared useful in differentiating *A*. *semotus* and *A*. *cinereus* (S5 Fig). Although the 2 species did not form reciprocally monophyletic lineages at this locus, the *A*. *cinereus* alleles were clearly different from the *A*. *semotus* alleles. It is not surprising that mtDNA does a better job at resolving recently diverged taxa because the theoretical effective population size of mtDNA is only ¼ that of biparentally inherited nuclear genes like CMA1. The relationships among *A*. *cinereus* and *A*. *semotus* CMA1 alleles were explored further by using allele networks (see below). CMA1 was also able to resolve relationships among most outgroup lasiurine species. The red bats (*Lasiurus*) and yellow bats (*Dasypterus*) are each strongly supported as monophyletic groups. The placement of *A*. *egregius* is not resolved, but the remaining hoary bats (*Aeorestes*) are strongly supported as monophyletic.

The Rag2 locus, on the other hand, was not able to differentiate among hoary bats nor between some of the outgroup lasiurine species (S6 Fig). Although there was divergence among alleles sequenced, the Rag2 locus did not separate *A*. *semotus*, *A*. *cinereus*, and *A*. *villosissimus*. Some yellow bat species (*Dasypterus*) had shared alleles at Rag2. Because the locus could not distinguish among several species that are otherwise well-differentiated (morphologically, geographically, and genetically at other loci), we do not believe that the inability to resolve relationships among hoary bats is due to extensive hybridization; rather, it is due to the resolving power of the locus itself. For this reason, we eliminated it from further analysis.

### Haplotype/Allele networks

The first CMA1 allele network (Fig 1), based on North American and Hawaiian bats, indicates that the 2 major groups of *A*. *semotus* alleles are independently derived from the *A*. *cinereu*s alleles. The network also indicates that there is a low level of “mismatching” in each major group. In other words, a few individuals with *A*. *cinereus* mtDNA haplotypes contain *A*. *semotus* nuclear alleles, and vice versa. These mismatched individuals are considered to have hybrid ancestry and this suggests that hybridization among *A*. *cinereus* and *A*. *semotus* has occurred; however, the network clearly shows that hybridization is not widespread. Another key finding is that there is no evidence of *A*. *semotus* alleles occurring in North America.

The second CMA1 allele network (Fig 1), based only on Hawaiian samples, depicts the geographic distribution of *A*. *cinereus* and *A*. *semotus* alleles on the archipelago. The island of Kauai was represented by 1 individual that contained only *A*. *semotus* alleles. The island of Maui contained both *A*. *cinereus* and *A*. *semotus* alleles. The island of Hawaii also contained both *A*. *cinereus* and *A*. *semotus* alleles. Previous studies already established that *A*. *cinereus* is present on Maui [1,2], but this is the first study to indicate that *A*. *cinereus* is present on Hawaii. One individual from the island of Hawaii (sample BPBM185538) was homozygous for *A*. *cinereus* alleles at CMA1 and had an *A*. *semotus* mtDNA haplotype. Russell et al. [2] also found evidence of *A*. *cinereus* on the island of Oahu; however, they did not sequence any Oahu specimens for CMA1 and so it remains unknown whether those individuals’ nuclear alleles match their mtDNA haplotype(s). Moreover, Russell et al. [2] only sequenced CMA1 for 2 individuals that were found to have an *A*. *cinereus* mtDNA haplotype. Both samples were from Maui and both had mismatched mtDNA haplotypes and CMA1 alleles (including one that is a potential F1 hybrid as it contained both *A*. *cinereus* and *A*. *semotus* alleles).

**Fig 2:**
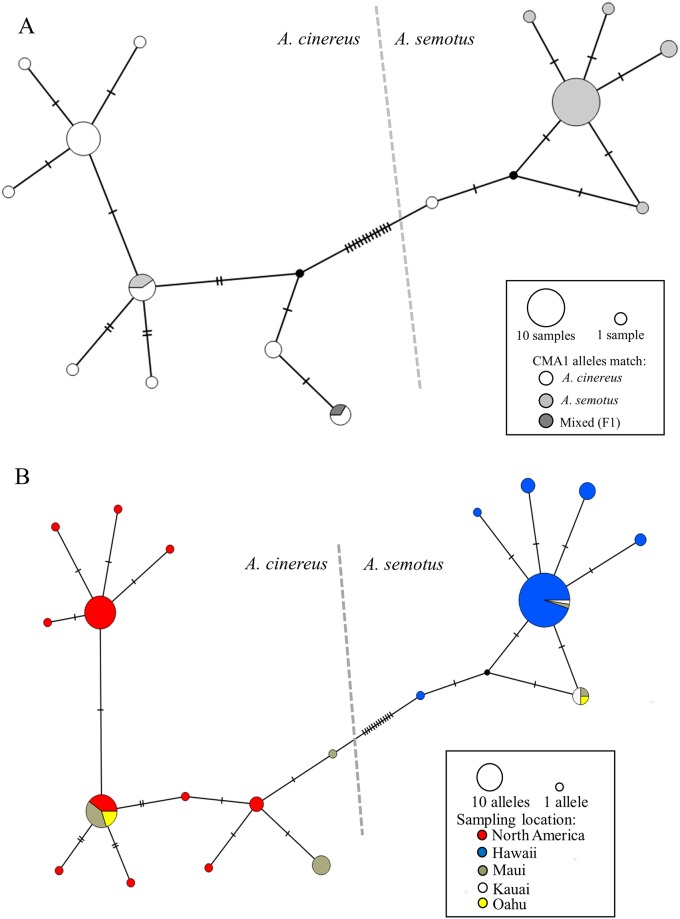
Haplotype networks based on mtDNA COI sequences. A) Network of haplotypes from North American and Hawaiian samples, colored to show the corresponding CMA1 alleles of individuals with the particular COI haplotype group (i.e. if the CMA1 alleles grouped with *A*. *semotus*, then it is colored light gray. If there was one allele each from *A*. *semotus* and *A*. *cinereus*, it is colored dark gray for “mixed.”). Only samples that were sequenced for both CMA1 and COI were included. B) Network of haplotypes from all North American and Hawaiian samples, colored by the geographic origin of the samples. Any discrepancies between parts A and B are due to the elimination of some samples in part A that were not sequenced for CMA1. Black dots represent inferred (unsampled) haplotypes.

The results of the COI haplotype networks further emphasized the results described above for CMA1. The first COI network (Fig 2) only contains samples for which CMA1 and COI were sequenced. Like the CMA1 allele network, it also shows a low level of mismatch among mtDNA haplotypes and nuclear alleles. The potential F1 hybrid is depicted as having “mixed” CMA1 alleles.

The second COI haplotype network (Fig 2B) is colored by the geographic origin of samples. Individuals of *A*. *cinereus* from the Hawaiian Islands contain 3 different COI haplotypes. One of those haplotypes is also shared by samples collected in North America; the other 2 haplotypes are unique to Hawaii (Island of Maui). Again, no evidence was found of North American samples with *A*. *semotus* haplotypes.

### Population historical demography

**Fig 3:**
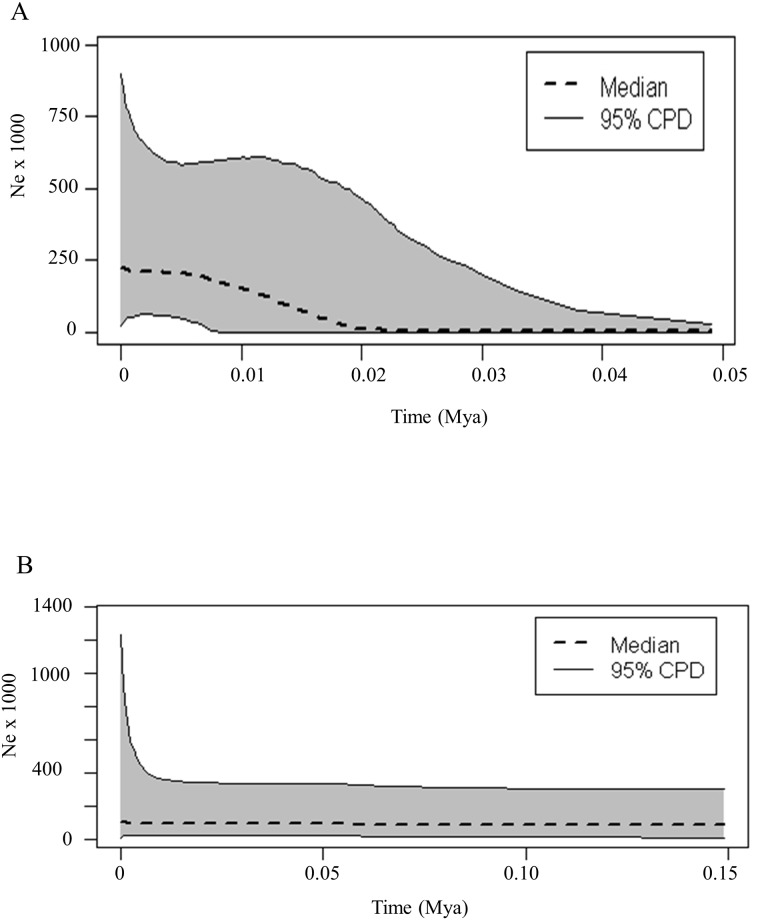
Extended Bayesian skyline plots based on combined CMA1 and COI sequences. A) Skyline plot for *A*. *semotus* only. B) Skyline plot for *A*. *cinereus* from the Hawaiian Islands only. Species classifications are based on mtDNA haplotype.

For scenario 1 (using *A*. *semotus* only), convergence was achieved after running 50 million generations in BEAST. Examination of the tree indicators in Tracer showed that a constant population through time could be rejected. Notable population expansion began approximately 20,000 years ago (Fig 3A).

For scenario 2 (*A*. *cinereus* on the Hawaiian Islands), convergence was achieved using 100 million generations. Examination of the tree indicators in Tracer showed that a hypothesis of constant population size through time could not be rejected. This is reflected in the EBSP (Fig 3) by the fact that the population size does not appear to change significantly through time.

### Divergence dating

**Fig 4:**
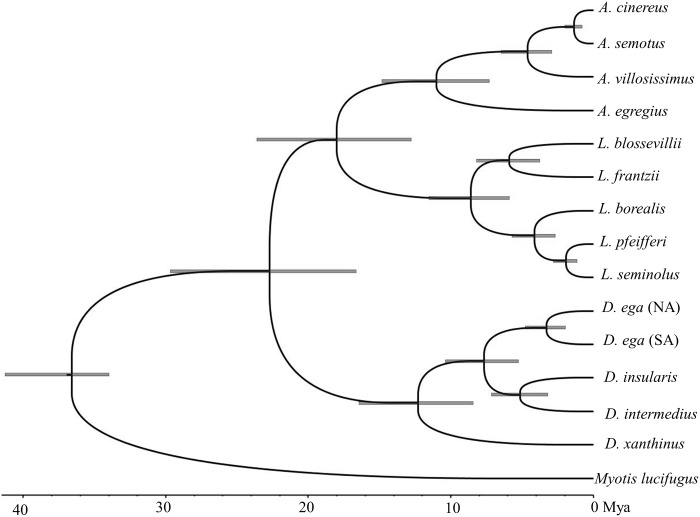
Divergence date estimates. The phylogeny is based on a Bayesian divergence time analysis from one individual of each species for 5 genes (COI, Cytb, ND1, ND2, and CMA1). Bars at nodes represent error estimates. All nodes were supported with a Bayesian posterior probability of 1.0. NA = North America; SA = South America.

The results from jModelTest for the reduced dataset used for divergence dating indicated that the appropriate models for each gene partition included K80+G for CMA1, HKY+I+G for COI, HKY+I+G for Cytb, GTR+I+G for ND1, and GTR+I+G for ND2. The results of divergence date estimates are shown in Fig 4. The dates from this analysis, which includes CMA1 nuclear data and mtDNA data using Cytb, COI, ND1, and ND2, largely correspond to the estimates presented in Baird et al. [1] based on mtDNA data alone and the recent estimates presented in Amador et al. [4] based on mtDNA and nuclear DNA. Here, the split between *Myotis* and the Lasiurini is estimated at approximately 36.58 Ma (34–41.28 Ma, 95% highest posterior density). The split between the yellow bats (*Dasypterus*) and the rest of Lasiurini was approximately 22.71 Ma (16.65–29.67 Ma). The split between the red (*Lasiurus*) and hoary bats (*Aeorestes*) occurred approximately 17.99 Ma (12.79–23.61 Ma). Within the genus *Aeorestes*, *A*. *villosissimus* diverged from *A*. *semotus* and *A*. *cinereus* approximately 4.61 Ma (2.93–6.47 Ma). *A*. *cinereus* and *A*. *semotus* are estimated to have diverged 1.35 Ma (0.79–1.98 Ma).

## Discussion

### Colonization of Hawaii

Phylogenetic data presented here agree with data presented in Baird et al. [1] that *A*. *semotus* represents an older colonization of the Hawaiian Islands, whereas *A*. *cinereus* colonized the Hawaiian Islands multiple times much more recently. These multiple, recent arrivals by *A*. *cinereus* are evidenced by the fact that 3 *A*. *cinereus* mtDNA haplotypes are found in the Hawaiian Islands, including 1 shared with North America and 2 endemic to the Hawaiian Islands. The Island of Maui contains the most *A*. *cinereus* diversity, with all 3 mtDNA haplotypes found there. We found 2 CMA1 alleles in *A*. *cinereus* on the Hawaiian Islands, 1 of which is present in multiple Hawaiian individuals and shared with multiple North American samples. The other is unique to one Hawaiian sample (which is a potential F1 hybrid as its other allele is an *A*. *semotus* allele).

Like Russell et al. [2], we found evidence for a relatively recent population expansion in *A*. *semotus* using EBSP. Data from Russell et al. [2] (based on COI, CMA1, and Rag2) indicated that the expansion began 10,000 years ago, but our data indicate that it began approximately 20,000 years ago.

We also examined the evolutionary history of *A*. *semotus* in the overall phylogenetic context of lasiurine bats. The phylogenetic dating analysis, which considers known fossil dates, places the divergence between *A*. *semotus* and *A*. *cinereus* at about 1.35 million years. Given that no *A*. *semotus* have been found in North America, we conclude that the divergence between *A*. *semotus* and *A*. *cinereus* is a result of the isolation of an *A*. *cinereus*-like relative that evolved into what we now know as *A*. *semotus* on the Hawaiian Islands post-colonization. An alternative explanation is that *A*. *semotus* diverged from *A*. *cinereus* in North America 1.35 Mya and subsequently colonized the Hawaiian Islands multiple times (bringing genetic diversity towhat is seen today on the islands) approximately 20,000 years ago at the time indicated by the population expansion visualized on the EBSP. For the alternative explanation to be true, it would necessitate the existence of a morphologically distinct and genetically diverse lineage (*A*. *semotus*) in North America for which no evidence has been found. It would also require multiple colonizationsof the Hawaiian Islands by the *A*. *semotus* lineage and its subsequent extinction in North America. It is unlikely that multiple colonizations of *A*. *semotus* from North America occurred around 20,000 years ago and more parsimonious to suggest that the extensive morphological, behavioral and genetic diversification of *A*. *semotus* took place in Hawaii, rather than North America, and over a period of >1,000,000 years.

Given the combination of data from EBSP and phylogenetics, we disagree with the conclusion of Russell et al. [2] that the population expansion represents the timing of colonization of the Hawaiian Islands. We interpret the EBSP results as simply an increase in population size about 20,000 years ago, not the initial founding of the population. The population size increase may be due to colonization of additional islands or other factors.

In the context of the geological history of the Hawaiian Islands, the hypothesis above seems logical. At our proposed colonization point of 1.3 Mya, the Island of Hawaii had not yet formed (it was formed approximately 0.43 Mya). Maui was relatively young (formed at least 1.3 Mya) and the other islands were well established, with the oldest (Kauai) being formed approximately 5 Mya [22,23]. Therefore, according to our timeline, *A*. *semotus* must initially have arrived on an island other than Hawaii and subsequently colonized the Island of Hawaii more recently, which may have led to the increased population size indicated by the EBSP. The colonization of the Island of Hawaii was subsequent to suitable habitat developing to support a population of bats on the island. The fact that Maui is where the highest diversity in hoary bats is observed may indicate that it is the oldest population. Further sampling is needed to test this hypothesis.

### Taxonomic status of hoary bats

Distinct monophyletic mtDNA lineages were observed between *A*. *semotus* and *A*. *cinereus* [1,2] (S1–S4 Figs). In this study, we show that *A*. *semotus* and *A*. *cinereus* have distinct CMA1 lineages. Although these lineages are paraphyletic, North American *A*. *cinereus* do not have *A*. *semotus* CMA1 alleles and the 2 species in Hawaii show only a low level of cross-specific allele sharing. Of the 45 hoary bats that were sequenced for both mtDNA and CMA1, only 4 individuals show mismatches where the lineage of their mtDNA haplotypes do not match the lineage of their CMA1 alleles. These animals are of potential hybrid ancestry. No potential hybrids were found among the North American hoary bats examined, all of which had only *A*. *cinereus* mtDNA haplotypes and CMA1 alleles. The 4 putative hybrids are among the 27 Hawaiian bats that were sequenced for both markers. Therefore, on the Hawaiian Islands the percentage of hybrids is <15%. Clearly these bats are not freely interbreeding to the extent one would expect if they represented members of the same species.

The fact that some hybridization occurs is not surprising if we accept that *A*. *semotus* evolved in isolation and diverged over a 1,000,000-year period with *A*. *cinereus* arriving very recently. Strong premating or postmating reproductive isolation mechanisms have not had time to become established. Moreover, some mammal species hybridize but maintain their species distinction, such as mule deer (*Odocoileus hemionus*) and white-tailed deer (*O*. *virginianus*). Several studies have examined hybridization between such species using a variety of genetic methods including molecular and biochemical markers, and biparentally, maternally and paternally inherited markers [24–31]. These studies confirmed that hybridization occurs and one study that looked at a 5-county area in Trans-Pecos Texas concluded that hybridization averaged 5.6% with a range of 0% to 13.8% in populations across this area [31]. This level of hybridization is comparable to that observed here between *A*. *semotus* and *A*. *cinereus* and we conclude that these 2 populations act as distinct species with some hybridization.

Russell et al. [2] hypothesized that because they did not find structure among *A*. *semotus* and *A*. *cinereus* (which they called “Hawaii1” and “Hawaii2”) at the nuclear loci (CMA1 and Rag2), that ongoing gene flow was occurring between the 2 populations. As discussed above, the Rag2 locus that they used was unable to differentiate even some outgroup taxa, and therefore is not an appropriate marker to use to differentiate these 2 species. Additionally, they only sequenced two individuals of *A*. *cinereus*-type specimens (defined based on mtDNA haplotype) for CMA1. With increased sequencing of Hawaiian bats, including additional *A*. *cinereus*-type bats, we have clearly demonstrated the distinction between *A*. *cinereus* and *A*. *semotus* at both mitochondrial and CMA1 nuclear loci.

Russell et al. [2] did not explicitly address the question of whether 2 extant species of hoary bats exist on the Hawaiian Islands. Their assumption was that *A*. *cinereus* and *A*. *semotus* represent different subspecies of the same species. They did not collect relevant data for testing the distinctiveness of these 2 (sub)species, especially by not sequencing nuclear CMA1 for all of their samples. No data were presented to test the differentiation between *A*. *cinereus* and *A*. *semotus* based on their nuclear data, such as a phylogeny or network for either of those loci (CMA1 and Rag2). Therefore, this study represents the most comprehensive test of the specific status of *A*. *cinereus* and *A*. *semotus* to date.

### Application of the name *A*. *semotus*

Ziegler et al. [8] objected to the application of the specific epithet “*semotus*” to the distinct, endemic Hawaiian form of hoary bats as proposed by Baird et al. [1]. They argued that since Baird et al. [1] did not examine the lectotype [32] of what was originally described as *Atalapha semota* Allen, 1890 [33], the name cannot be assigned to a lineage with certainty, and so it should not be used.

Although it would certainly be useful to examine the genetics of the *A*. *semotus* lectotype, it is difficult to obtain useful genetic samples from such specimens without specialized techniques and facilities. Lacking these, we cannot assign the lectotype to a particular lineage with absolute certainty and will not risk destructive sampling to the lectotype without a high probability of obtaining good data. Therefore, we continue to support the most reasonable conclusion that the morphologically, behaviorally, and genetically distinct lineage (endemic to Hawaii) should be called *A*. *semotus*. As with all taxonomic classifications, this is a hypothesis open to further testing. The alternatives to this application of the name *A*. *semotus* are to either: 1) not recognize the taxonomic diversity present between the Hawaiian and North America forms (i.e. leaving them as members of the same species) or 2) assign *A*. *semotus* as a junior synonym to *A*. *cinereus* and erect a completely new name for the Hawaiian hoary bats. We believe that option 1 would not do justice to the clear species-level differences present between the hoary bats that we have demonstrated. Option 2 would cause more confusion than the taxonomic arrangement proposed by Baird et al. [1]. We welcome further studies that include the lectotype to clarify its relationship to the genetic lineages of hoary bats.

Part of the opposition by Ziegler et al. [8] to assigning the name “*semotus*” to the Hawaiian lineage without use of the lectotype is that the type locality (specific island) is not known [32] and since the two putative species co-occur broadly across many islands it is possible that both occur on the island from which the lectotype originated. However, they mis-state the distribution where both hoary bat lineages occur. They claim that both co-occur on the islands of Maui, Kauai, and Oahu “suggesting that both groups may occur in sympatry broadly across the islands,” citing Russell et al. [2] and Baird et al. [1] for this claim. In fact, these papers both state that the two hoary bat forms co-occur on Maui and Oahu. The present paper shows additional data for one individual of hybrid ancestry co-occurring with *A*. *semotus* on the Island of Hawaii (no “pure” *A*. *cinereus* have been found on the Island of Hawaii). Thus, there is no data showing co-occurrence on Kauai and very little evidence of potential co-occurrence on Hawaii, certainly not evidence for broad sympatry across all the islands. *A*. *cinereus* has been sampled less frequently than *A*. *semotus* (Table 1), perhaps indicating that the former is less common. Although the specific island for the lectotype is unknown, another specimen with the same collector reported in the original description by Allen [33] originated from Kauai. We note that to date, only *A*. *semotus* is known from Kauai, although with limited sampling from that island.

### Generic-level taxonomy in Lasiurini

The analyses and data in this paper, including the phylogenetic analysis and dating based on mtDNA and the nuclear CMA1 gene, support the findings of Baird et al. [1] that the red, yellow, and hoary bats are genetically highly divergent. Although Amador et al. [4] found *D*. *intermedius* as the sister taxon to the red bats (see S6 Fig in [4]), it was with relatively low levels of support. With increased taxon sampling, we consistently find *D*. *intermedius* included within the yellow bats with very high support. This finding is more consistent with the morphology of *D*. *intermedius* as a yellow bat, rather than its placement with the red bats as in Amador et al. [4]. In light of all recent molecular data [(this study, [1, 4, 34, 35]), we recommend continued use of the genus names *Lasiurus* (red bats), *Dasypterus* (yellow bats) and *Aeorestes* (hoary bats, including *A*. *egregius*) as proposed in Baird et al. [1].

There are several reasons to support this recommendation. First, striking morphological differences exist among the 3 groups. The 3 genera have long been easily distinguishable and referred to colloquially by the terms “red,” “yellow,” and “hoary bats.” In contrast, many other vespertilionid genera must be closely examined to find morphological differences, such as the number of teeth. Of course, the absence of distinct and visible morphological differences among some genera does not mean that they are not valid as distinct genera; however, the presence of such striking morphological differences among the lasiurine genera emphasizes the relative magnitudes of these differences in comparison to other vespertilionids. Second, the morphological divergence is well reflected in all the available genetic data, including allozymes [36], and mitochondrial [1,37] and nuclear DNA sequences ([1], this study). In comparison with other chiropterans, there are higher levels of genetic divergence among the 3 lasiurine genera than are typical of inter-generic differences among bat species [38]. Baker and Bradley [38] report average inter-generic differences of bats at 12.0%. We found differences of 18.79% (yellow to hoary bats), 19.05% (hoary to red bats), and 19.79% (yellow to red bats; data not shown but derived from values in Table 3). Third, the taxonomy of lasiurine bats has changed many times since its original description. Red, yellow, and hoary bats have each been classified as a distinct genus in the past [39,40,41] (see complete synonymy in [1]). Immediately prior to Baird et al. [1], they had been recognized as members of the same genus (*Lasiurus*). Nevertheless, we consider that the proposed changes are the best reflection of all available data, including morphological and molecular data. Our aim by recommending these changes was to provide a taxonomy that acknowledges the uniqueness of each genus and that will lead to taxonomic stability.

### Response to Ziegler et al. [8]

The proposed taxonomic changes included in Baird et al. [1], and supported here, are not necessarily novel. Red, yellow, and hoary bats have previously been considered separate genera; Hawaiian, South American, and North American hoary bats all were originally described as distinct species. Nonetheless, Ziegler et al. [8] rejected the taxonomic changes proposed in Baird et al. [1]. Regarding the changes to hoary bat taxonomy, specifically the elevation of *A*. *semotus* to species status, they state that “convincing evidence that Hawaiian populations represent a distinct species (rather than a subspecies of *L*. [*A*.] *cinereus*) has been lacking.” To support this claim, they cite the findings of Russell et al. [2], specifically the conclusion regarding the 10,000 year estimate of time since divergence from North American *A*. *cinereus* (which, as discussed above, we believe to be an incorrect interpretation of the data). They also cite the lack of differentiation reported at nuclear loci, but do note the high degree of mitochondrial differentiation at COI (~3%). However, they give an inaccurate divergence estimate of Cytb among North American and Hawaiian lineages from Baird et al. [1], at 2%, when in fact it is 4% divergence (page 1262; see also Table 3 depicting Cytb divergence in this study). The correct level of Cytb divergence (4.2%; Table 3) exceeds the typical intraspecific divergence in bats reported by Baker and Bradley [38], which Ziegler et al. [8] themselves cite as precedent for describing species-level differentiation in mammals. The present paper provides new data and analyses that supports elevating hoary bats to specific status. Phylogenetic analyses of mtDNA and CMA1 are shown to differentiate between *A*. *cinereus* and *A*. *semotus* (and other lasiurine bats) and the small number of mismatches of mtDNA and CMA1 show restricted levels of hybridization, despite claims to the contrary by Russell et al. [2]. We also demonstrate here that Rag2, given its inability to resolve otherwise easily distinguishable species, was not a useful locus for testing hypotheses of species status in hoary bats.

Ziegler et al. [8] appear to acknowledge that mammalian species can be distinguished by the observation of high levels of genetic divergence. Their error regarding the levels of divergence reported in Baird et al. [1] appears to be their only argument for not recognizing the distinction of Hawaiian hoary bats. They cite examples of morphological and behavioral differences between Hawaiian and North American hoary bats, such as Hawaiian hoary bats being smaller in size, having a proportionally larger gape and masseter muscles [42], and higher frequency echolocation calls [43,44]. Hawaiian hoary bats also roost in caves, whereas North American hoary bats roost in trees [45] and have a more generalized diet [46]. Most of the studies cited above [42, 45, 46] only examined bats from the Island of Hawaii, where the clear majority of samples are *A*. *semotus*. We have only found 1 hybrid individual and no pure *A*. *cinereus* on the island of Hawaii. Therefore, most studies showing differences between mainland and Hawaiian hoary bats most likely included only *A*. *semotus* on Hawaii, which we note is a distinct species from *A*. *cinereus*. It remains to be seen whether *A*. *cinereus* on the Hawaiian Islands have morphological and behavioral traits more similar to mainland *A*. *cinereus* or *A*. *semotus*.

Ziegler et al. [8] objected to the taxonomic changes in Lasiurini proposed by Baird et al. [1] on the grounds that “well-established zoological nomenclature” will be disrupted by the changes. As evidence, they state that a Google Scholar search resulted in a high number of publications using “*Lasiurus cinereus*.” By definition, taxonomic change to any recognized taxonomic unit will have the effect of changing previously established nomenclature, as has been shown for reptiles, amphibians and other groups (e.g. [47,48]). The argument of using traditional taxonomy for tradition’s sake does not outweigh the new scientific evidence that Lasiurini is most appropriately divided into 3 genera and that the 3 hoary bat lineages are distinct species. Amador et al. [4], in their thorough investigation of bat systematics stated that their results “supported the recent generic splits of Baird et al. [1].”

Ziegler et al. [8] also argue that, while *Aeorestes* is an available name for hoary bats [49], it should not be used for hoary bats because it was previously used (incorrectly) by other authors as the name of a subgenus of *Myotis*. According to the rules of zoological nomenclature, “the valid name of a taxon is the oldest available name applied to it…” (Article 23.1, ICZN), which in this case is *Aeorestes*. Although the code allows for the Principle of Priority to be set aside in certain cases, we do not believe it is necessary to violate that Principle in this case. Ziegler et al. [8] argue against the use of this genus name because it will cause “extensive confusion” due to its previous incorrect usage; however, they state that they found only 10 papers since 1900 that have included the name. We doubt that the use of an incorrect name in 10 papers over 117 years will cause much confusion.

In their opposition to the generic-level changes to lasiurine taxonomy, Ziegler et al. [8] argue that “separate generic epithets for the 3 major lineages within *Lasiurus* sensu lato are not required to keep any taxon monophyletic since all workers agree that *Lasiurus* sensu lato is clearly monophyletic.” This is perhaps their most logical argument against taxonomic changes, and one that the authors of Baird et al. [1] carefully weighed before proposing these changes. We agree that *Lasiurus* sensu lato was monophyletic. In many cases, we also agree that breaking up an otherwise monophyletic group without clear, convincing reasons is unwarranted. However, in this case there are clear reasons to do so. The proposed changes more accurately reflect the deep genetic and morphological distinction among the proposed genera. Additionally, the tribe-level taxonomy, which has never changed, still indicates that *Aeorestes*, *Dasypterus*, and *Lasiurus* together form a monophyletic tribe, Lasiurini, which is highly distinct from all other vespertilionids. Genera should be monophyletic groups, and the newly applied *Aeorestes*, *Dasypterus*, and *Lasiurus* are each monophyletic. It is deciding the scale of monophyly that should apply to a single genus that is the tricky question.

Using subgenera, rather than elevating red, yellow, and hoary bats to separate genera, was proposed by Ziegler et al. [8]. This proposal is an alternative way to describe the distinction among the 3 lineages; however, a subgenus distinction is almost never utilized in the literature and would quickly become obsolete. Therefore, using subgeneric names would not solve the problem of having taxonomy under-represent the distinction among red, yellow, and hoary bats. In the past, when all 3 groups were considered members of the genus *Lasiurus*, literature that referred to that genus was ambiguous as to whether the study included red, yellow, or hoary bats (or multiple groups) without additional information. With the revised taxonomy, literature searches will become clearer as to which groups are being studied.

A key point to consider with the science of taxonomy is its broader uses, other than simply assigning names. Taxonomy can and should inform us about both evolution and biodiversity. With the revision to lasiurine taxonomy proposed by Baird et al. [1], that utility of taxonomy is maximized. The revised lasiurine taxonomy more accurately reflects deep morphological and genetic diversity within the tribe at the generic level. The changes to the hoary bat taxonomy better reflect our current understanding of the morphological, genetic, behavioral, and acoustic differentiation between the species. Data presented here show that the different hoary bats are not interbreeding to the degree one would expect of members of the same species. All of the genetic data we have examined support these changes, in addition to long-established morphological differences among the genera.

### Conservation implications

Fully understanding the relationships and taxonomy of the hoary bats has important conservation implications. Currently, the conservation status of Hawaiian hoary bats reflects the previous taxonomy: *Lasiurus cinereus semotus* is considered endangered; *L*. *c*. *cinereus* is not. The conservation status of *A*. *cinereus* needs to be revisited due to the documentation of that species on the Hawaiian Islands. Populations of *A*. *cinereus* on the Hawaiian archipelago should be considered for endangered status. If we simply retained the previous taxonomy and called the different genetic groups “Hawaii1” and “Hawaii2” as Russell et al. [2] termed them, we would not be doing justice to the diversity and potential conservation issues in Hawaii. While the EBSP shows an estimate of effective population size (*N*_*e*_), we caution that the estimate shown in Fig 3 does not necessarily correspond to the actual population size. The estimate of *N*_*e*_ for the EBSP is highly dependent on the assumed generation time, for which we have followed Russell et al. [2] by using a generation time of 2 years. The accuracy of that generation time should be examined further if having a more precise estimate of *N*_*e*_ is desired. As this paper makes clear, there are only two extant species of terrestrial mammals native to Hawaii, and very likely both require special conservation attention.

## Supporting information

S1 Fig Bayesian phylogeny based on mitochondrial ND1 sequences. Numbers at nodes represent Bayesian posterior probabilities.
(PDF)

S2 Fig Bayesian phylogeny based on mitochondrial ND2 sequences. Numbers at nodes represent Bayesian posterior probabilities.
(PDF)

S3 Fig Bayesian phylogeny based on mitochondrial cytb sequences. Numbers at nodes represent Bayesian posterior probabilities.
(PDF)

S4 Fig Bayesian phylogeny based on mitochondrial COI sequences. Numbers at nodes represent Bayesian posterior probabilities.
(PDF)

S5 Fig Bayesian phylogeny based on nuclear CMA1 sequences. Individual alleles were used in the analysis. Numbers at nodes represent Bayesian posterior probabilities. Letters following sample names indicate alleles: “A” and “B” represent two different alleles from the same specimen; “AA” represents a homozygous specimen.
(PDF)

S6 Fig Bayesian phylogeny based on nuclear Rag2 sequences. Where available, individual alleles were used in the analysis. Numbers at nodes represent Bayesian posterior probabilities. Letters following sample names indicate alleles: “A” and “B” represent two different alleles from the same specimen; “AA” represents a homozygous specimen; “UR” represents unresolved alleles (in this case, the consensus sequence of the two alleles was used in the analysis).
(PDF)

## References

[1] Baird AB, Braun JK, Mares MA, Morales JC, Patton JC, Tran CQ, et al Molecular systematic revision of tree bats (Lasiurini): doubling the native mammals of the Hawaiian Islands. J Mammal 2015; 96:1255–1274.

[2] Russell AL, Pinzari CA, Vonhof MJ, Olival KJ, Bonaccorso FJ. Two tickets to paradise: multiple dispersal events in the founding of hoary bat populations in Hawai'i. PLoS ONE 2015; 10: e0127912; doi: 10.1371/journal.pone.0127912 26083029 10.1371/journal.pone.0127912 PMC4471086

[3] Nabholz B, Glémin S, Galtier N. The erratic mitochondrial clock: variations of mutation rate, not population size, affect mtDNA diversity across birds and mammals. BMC Evol Biol 2009:54; doi: 10.1186/1471-2148-9-54 19284537 10.1186/1471-2148-9-54 PMC2660308

[4] Amador LL, Moyers Arevalo RL, Almeida FC, Catalano SA, Giannini NP. Bat systematics in the light of unconstrained analyses of a comprehensive molecular supermatrix. J Mamm Evol 2016 doi: 10.1007/s10914-016-9363-8

[5] Giménez AL, Giannini NP. The endemic Patagonian vespertilionid assemblage is a depauperate ecomorphological vicariant of species-rich Neotropical assemblages. Curr Zool 2016; zow100. 10.1093/cz/zow100 PMC5804208 29492009

[6] Rodríguez-San Pedro A, Allendes JL. *Lasiurus borealis* (Müller, 1776): una especie erróneamente reconocida dentro de la quiropterofauna de Chile. Biodiversity and Natural History 2016; 2:10–12.

[7] Schmidly DJ, Bradley RD. The Mammals of Texas (7th Edition). Austin: University of Texas Press; 2016.

[8] Ziegler AC, Howarth FG, Simmons NB. A second endemic land mammal for the Hawaiian Islands: a new genus and species of fossil bat (Chiroptera: Vespertilionidae). Am Mus Novit 2016; 3854:1–52.

[9] Baird AB, Hillis DM, Patton JC, Bickham JW. Speciation by monobrachial centric fusions: A test of the model using nuclear DNA sequences from the bat genus *Rhogeessa*. Mol Phylogenet Evol 2009; 50: 256–267. doi: 10.1016/j.ympev.2008.11.001 19038350 10.1016/j.ympev.2008.11.001

[10] Kearse M, Moir MR, Wilson A, Stones-Havas S, Cheung M, Sturrock S, et al Geneious Basic: an integrated and extendable desktop software platform for the organization and analysis of sequence data. Bioinformatics 2012; 28: 1647–1649. doi: 10.1093/bioinformatics/bts199 22543367 10.1093/bioinformatics/bts199 PMC3371832

[11] Darriba D, Taboada GL, Doallo R, Posada. jModelTest 2: More models, new heuristics and parallel computing. Nat Methods 2012; 9: 772. 10.1038/nmeth.2109 PMC4594756 22847109

[12] Guindon S, Gascuel O. A simple, fast and accurate method to estimate large phylogenies by maximum-likelihood. Syst Biol 2003; 52: 696–704. 14530136 10.1080/10635150390235520

[13] Huelsenbeck JP, Ronquist F. MRBAYES: Bayesian inference of phylogeny. Bioinformatics 2001; 17: 754–755. 11524383 10.1093/bioinformatics/17.8.754

[14] Ronquist F, Huelsenbeck JP. MRBAYES 3: Bayesian phylogenetic inference under mixed models. Bioinformatics 2003; 19: 1572–1574. 12912839 10.1093/bioinformatics/btg180

[15] Rambaut A, Drummond AJ. 2009. Tracer v 1.5. Retrieved from http://beast.bio.ed.ac.uk/. Accessed 28 June 2016.

[16] Rambaut A. 2012. FigTree v 1.4. http://beast.bio.ed.ac.uk/. Accessed 28 October 2013.

[17] Clement M, Snell Q, Walke P, Posada D. Crandall K. TCS: estimating gene genealogies. Proceedings of the 16th International Parallel and Distributed Processing Symposium 2002; 2: 184.

[18] Bouckaert R, Heled J, Kuhnert D, Vaughan T, Wu C-H, Xie D, et al BEAST 2: A software platform for Bayesian evolutionary Analysis. PLoS Comput Biol 2014; 10: e1003537 doi: 10.1371/journal.pcbi.1003537 24722319 10.1371/journal.pcbi.1003537 PMC3985171

[19] Heled J, Drummond AJ. Bayesian inference of population size history from multiple loci. BMC Evol Biol 2008; 8: 289 doi: 10.1186/1471-2148-8-289 18947398 10.1186/1471-2148-8-289 PMC2636790

[20] Gunnell GF, Simons EL, Seiffert ER. New bats (Mammalia: Chiroptera) from the Late Eocene and Early Oligocene, Fayum Depression, Egypt. J Vertebr Paleontol 2008; 28:1–11.

[21] Eiting TP, Gunnell GF. Global completeness of the bat fossil record. J Mamm Evol 2009; 16:151–173.

[22] Fleischer RC, McIntosh CE, Tarr CL. Evolution on a volcanic conveyor belt: using phylogeographic reconstructions and K-Ar-based ages of the Hawaiian Islands to estimate molecular evolutionary rates. Mol Ecol 1998; 7: 533–545. 9628004 10.1046/j.1365-294x.1998.00364.x

[23] Naughton JJ, Macdonald GA, Greenberg VA. Some additional potassium-argon ages of Hawaiian rocks: The Maui volcanic complex of Molokai, Maui, Lanai and Kahoolawe. Journal of Volcanology and Geothermal Research 1980; 7: 339–355.

[24] Ballinger SW, Blakenship LH, Bickham JW, Carr SM. Allozyme and mitochondrial DNA analysis of a hybrid zone between white-tailed deer and mule deer (*Odocoileus*) in West Texas. Biochemical Genetics 1992; 30:1–11. 1325774 10.1007/BF00554423

[25] Bradley RD, Baker RJ. A test of the genetic species concept: cytochrome-b sequences and mammals. J Mammal 2001; 82:960–973. 10.1644/06-MAMM-F-038R2.1 PMC2771874 19890476

[26] Carr SM, Ballinger SW, Derr JN, Blakenship LH, Bickham JW. Mitochondrial DNA analysis of hybridization between sympatric white-tailed deer and mule deer in West Texas. Proc Natl Acad Sci U S A 1986; 83:9576–9580. 3467326 10.1073/pnas.83.24.9576 PMC387183

[27] Carr SM, Hughes GA. Direction of introgressive hybridization between species of North American deer (*Odocoileus*) as inferred from mitochondrial-cytochrome b sequences. J Mammal 1993; 74:331–343.

[28] Cathey JC, Bickham JW, Patton JC. Introgressive hybridization and nonconcordant evolutionary history of maternal and paternal lineages in North American deer. Evolution 1998; 52: 1224–1229. doi: 10.1111/j.1558-5646.1998.tb01850.x 28565226 10.1111/j.1558-5646.1998.tb01850.x

[29] Cronin MA, Vyse Er, Cameron DG. Genetic relationships between mule deer and white-tailed deer in Montana. J Wildl Manage 1988; 52:320–328.

[30] Derr JN. Genetic interactions between whitetailed and mule deer in the southwestern United States. J Wildl Manage 1991; 55:228–237.

[31] Stubblefield SS, Warren RJ, Murphy BR. Hybridization of free-ranging white-tailed and mule deer in Texas. J Wildl Manage 1986; 50:688–690.

[32] Lyon MW, Osgood WH. Catalogue of the type specimens of mammals in the United States National Museum, including the Biological Survey Collection. Bull United States Natl Mus 1909; 62: 1–325.

[33] Allen H. Description of a new species of bat, *Atalapha semota*. Proc United States Natl Mus 1890; 13: 173–175.

[34] Hoofer SR, Van Den Bussche RA. Molecular phylogenetics of the chiropteran family Vespertilionidae. Acta Chiropt 2003; 5:1–63.

[35] Roehrs ZP, Lack JB, Van Den Bussche RA. Tribal phylogenetic relationships within Vespertilioninae (Chiroptera: Vespertilionidae) based on mitochondrial and nuclear sequence data. J Mammal 2010; 91:1073–1092.

[36] Baker RJ, Patton JC, Genoways HH, Bickham JW. Genic studies of *Lasiurus* (Chiroptera: Vespertilionidae). Occ Pap Tex Tech Univ Mus 1988; 117: 1–15.

[37] Morales JC, Bickham JW. Molecular systematics of the genus *Lasiurus* (Chiroptera: Vespertilionidae) based on restriction-site maps of the mitochondrial ribosomal genes. J Mammal 1995; 76: 730–749.

[38] Baker RJ, Bradley RD. Speciation in mammals and the genetic species concept. J Mammal 2006; 87:643–662. doi: 10.1644/06-MAMM-F-038R2.1 19890476 10.1644/06-MAMM-F-038R2.1 PMC2771874

[39] Fitzinger LJ. Kritische durchsicht der ordnung der flatterthiere oder handflüger (Chiroptera). Sitzungsberichte der Mathematisch-Naturwissenschaftlichen Classe der Kaiserlichen Akademie der Wissenschaften 1870; 62:353–438.

[40] Peters W. Eine monographische übersicht der chiropterengattungen *Nycteris* und *Atalapha* vor. Monatsberichte der Königlich Preussischen Akademie der Wissenschaften zu Berlin 1871: 900–914.

[41] Tate GHH. Review of the vespertilionine bats, with special attention to the genera and species of the Archbold collections. Bull Am Mus Nat Hist 1942; 80: 221–297.

[42] Jacobs D. Morphological divergence in an insular bat, *Lasiurus cinereus semotus*. Funct Ecol 1996; 10:622–630.

[43] Barclay RMR. The echolocation calls of hoary (*Lasiurus cinereus*) and silver-haired (*Lasionycteris noctivagans*) bats as adaptations for long- versus short-range foraging strategies and the consequences for prey selection. Can J Zool 1986; 64:2700–2705.

[44] Barclay RMR, Fullard JH, Jacobs DS. Variation in the echolocation calls of the hoary bat (*Lasiurus cinereus*): influence of body size, habitat structure, and geographic location. Can J Zool 1999; 77:530–534.

[45] Fujioka K, Gon S. Observations of the Hawaiian Bat (*Lasiurus cinereus semotus*) in the Districts of Ka'ū and South Kona, Island of Hawai'i. J Mammal 1988; 69:369–371.

[46] Whitaker JO Jr., Tomich PQ. Food habits of the hoary bat, *Lasiurus cinereus*, from Hawaii. J Mammal 1983; 64:151–152.

[47] Pyron RA, Burbrink FT, Weins JJ. A phylogeny and revised classification of Squamata, including 4161 species of lizards and snakes. BMC Evol Biol 2013;13:93 doi: 10.1186/1471-2148-13-93 23627680 10.1186/1471-2148-13-93 PMC3682911

[48] Frost DR, Grant T, Faivovich J, Bain RH, Haas A, Haddad CFB, et al The amphibian tree of life. Bull Am Mus Nat Hist 2006; 297: 1–291.

[49] Gardner AL, Handley CO Jr. Genus *Lasiurus* Pp. 457–468 in Mammals of South America. Volume 1: marsupials, xenarthrans, shrews, and bats (Gardner A. L., ed.). Chicago: University of Chicago Press; 2007.

