# Supplementary figures and images for: Nuclear and mtDNA phylogenetic analyses clarify the evolutionary history of two species of native Hawaiian bats and the taxonomy of Lasiurini (Mammalia: Chiroptera)
Source: PLoS One. 2017 Oct 11;12(10):e0186085. doi: 10.1371/journal.pone.0186085 (PMC5636129; doi:10.1371/journal.pone.0186085)

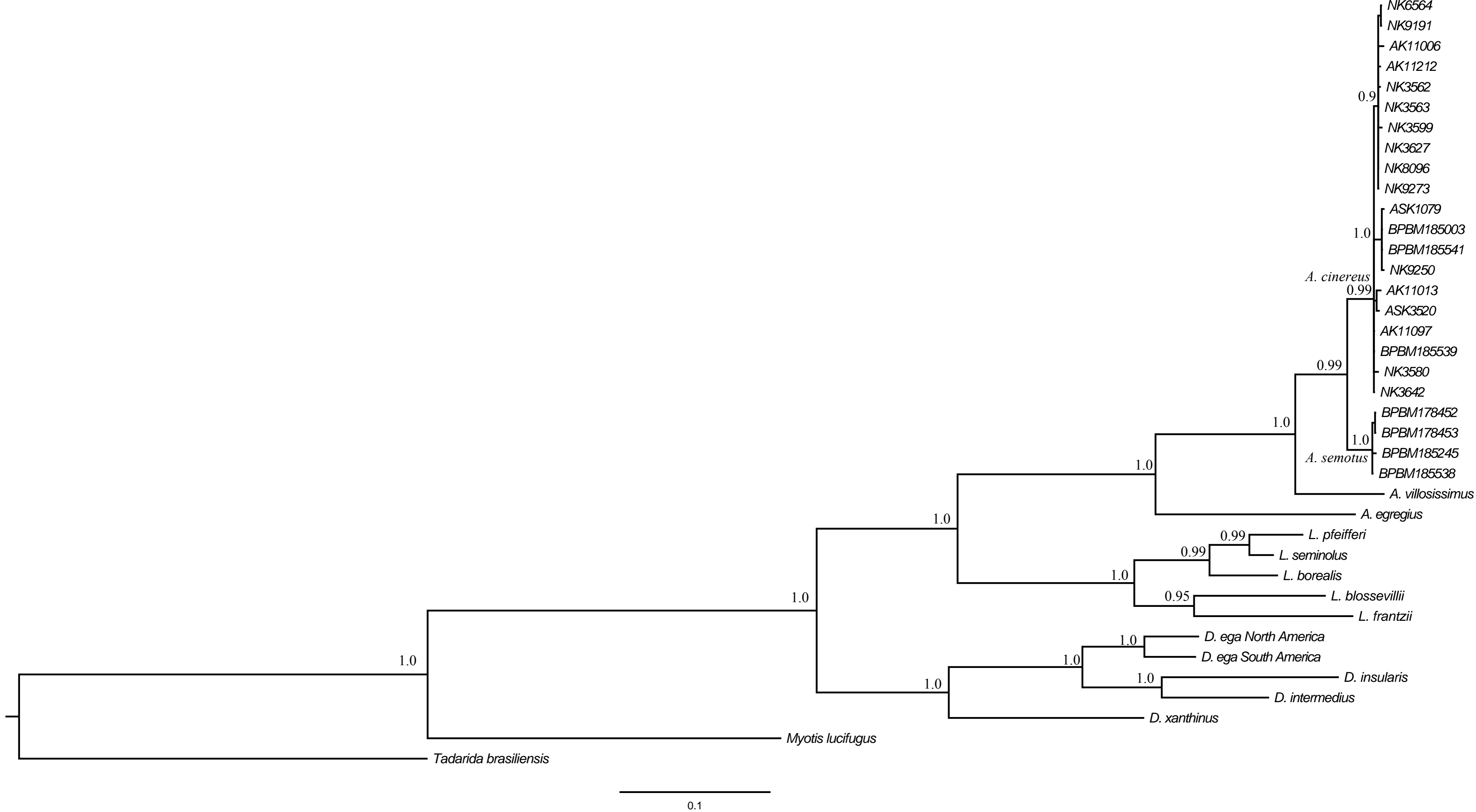

Supplement: S1 Fig — Numbers at nodes represent Bayesian posterior probabilities. (PDF) [file pone.0186085.s001.pdf]

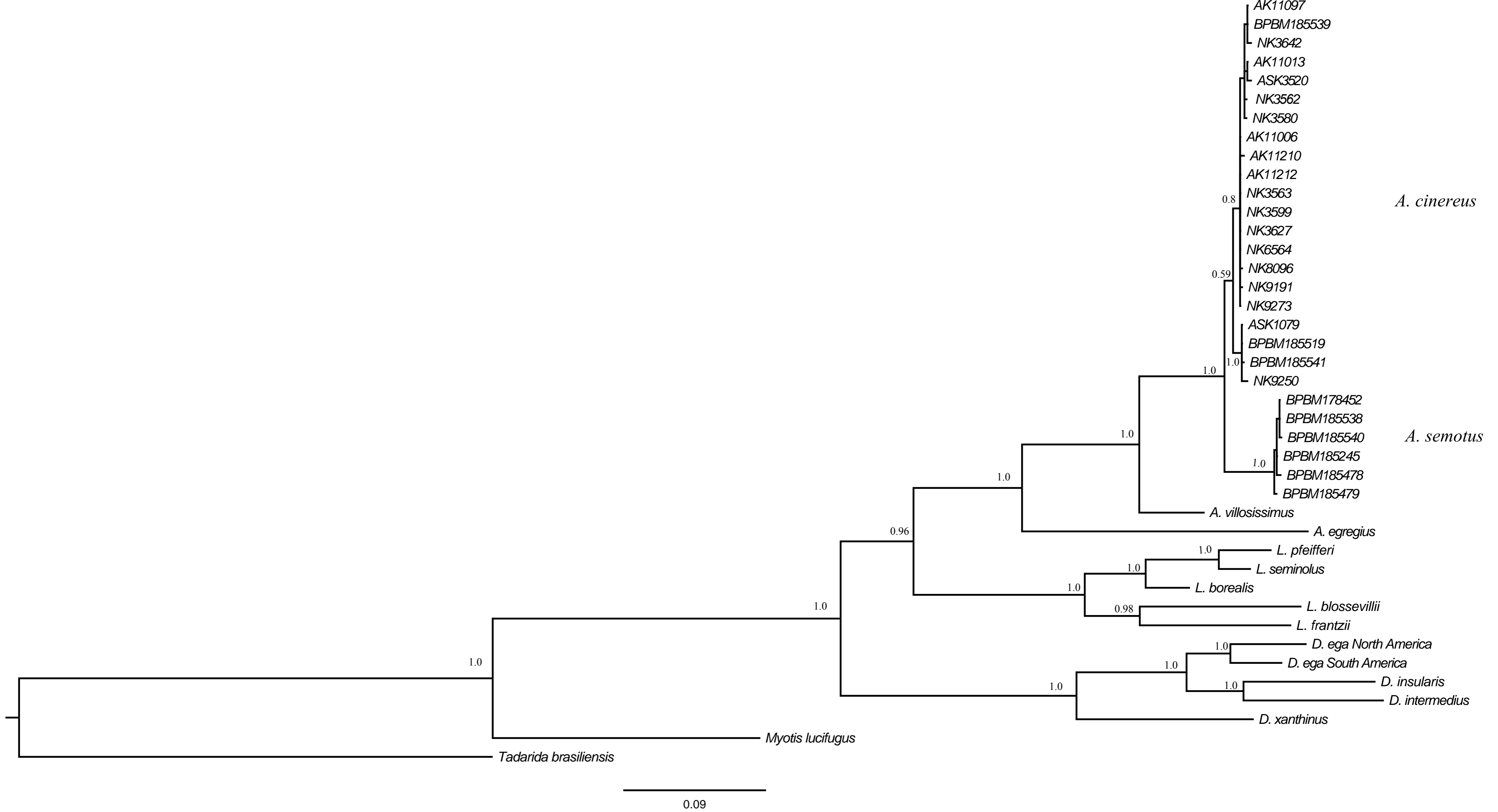

Supplement: S2 Fig — Numbers at nodes represent Bayesian posterior probabilities. (PDF) [file pone.0186085.s002.pdf]

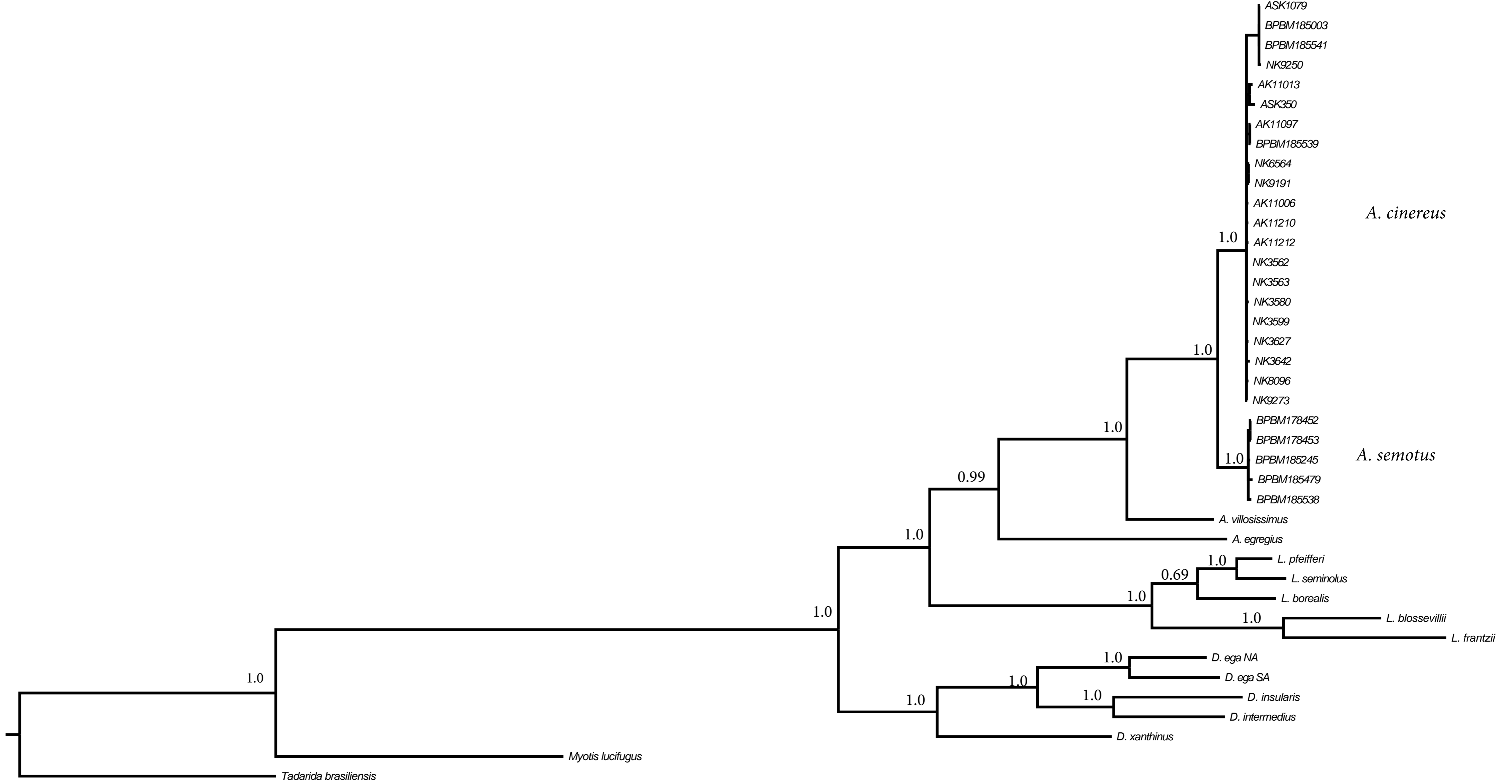

0.2

Supplement: S3 Fig — Numbers at nodes represent Bayesian posterior probabilities. (PDF) [file pone.0186085.s003.pdf]

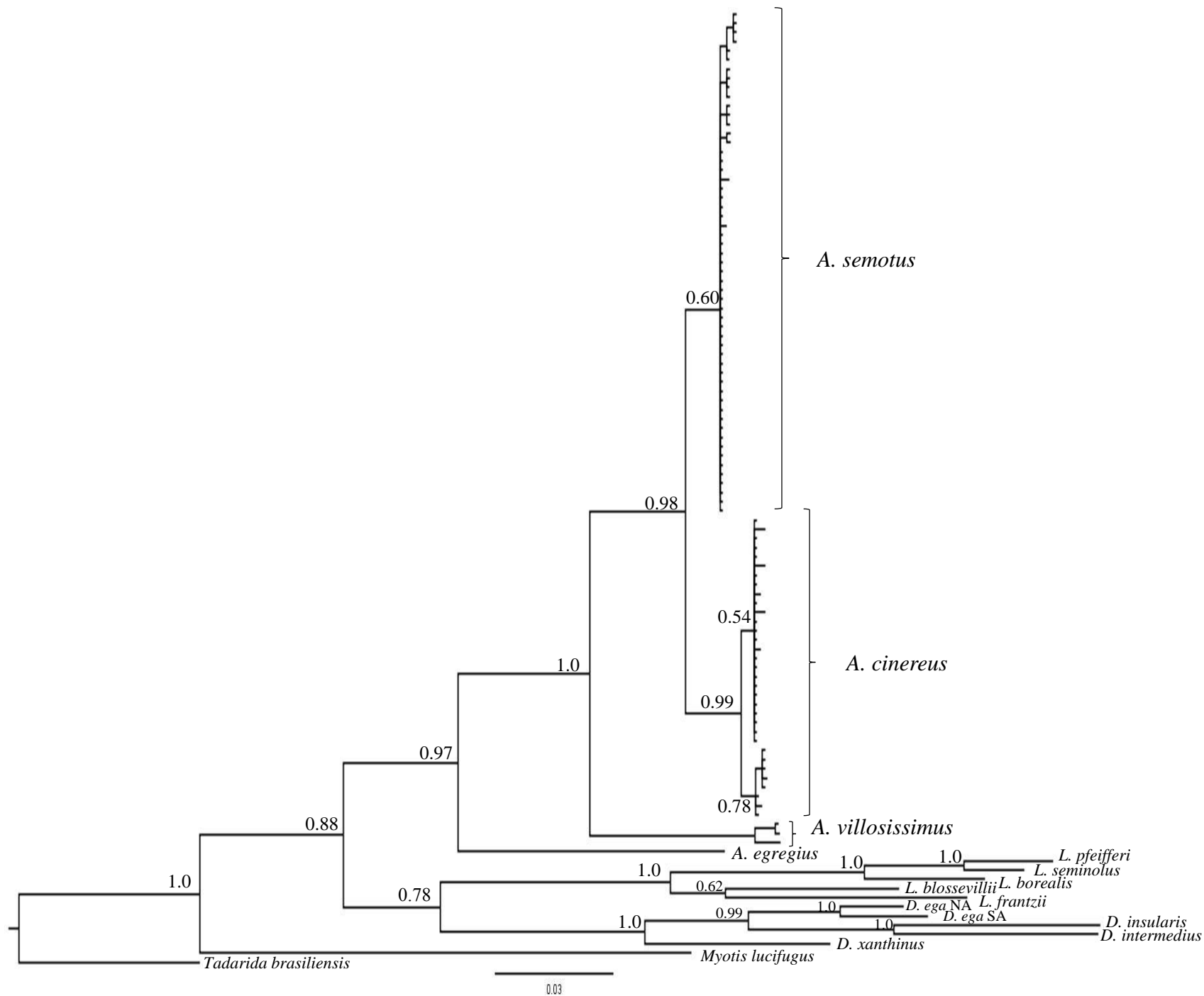

Supplement: S4 Fig — Numbers at nodes represent Bayesian posterior probabilities. (PDF) [file pone.0186085.s004.pdf]

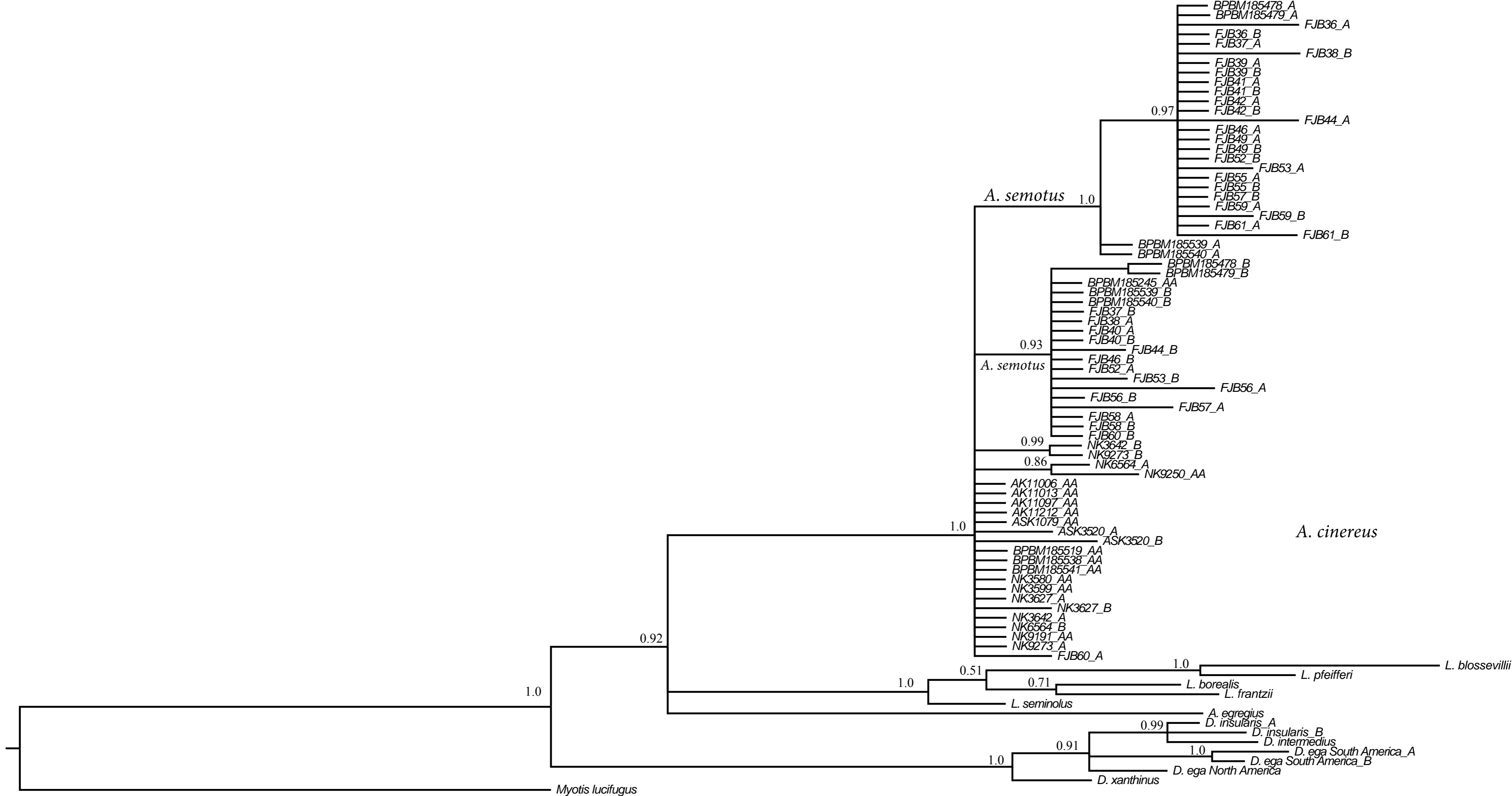

0.002

Supplement: S5 Fig — Individual alleles were used in the analysis. Numbers at nodes represent Bayesian posterior probabilities. Letters following sample names indicate alleles: “A” and “B” represent two different alleles from the same specimen; “AA” represents a homozygous specimen. (PDF) [file pone.0186085.s005.pdf]

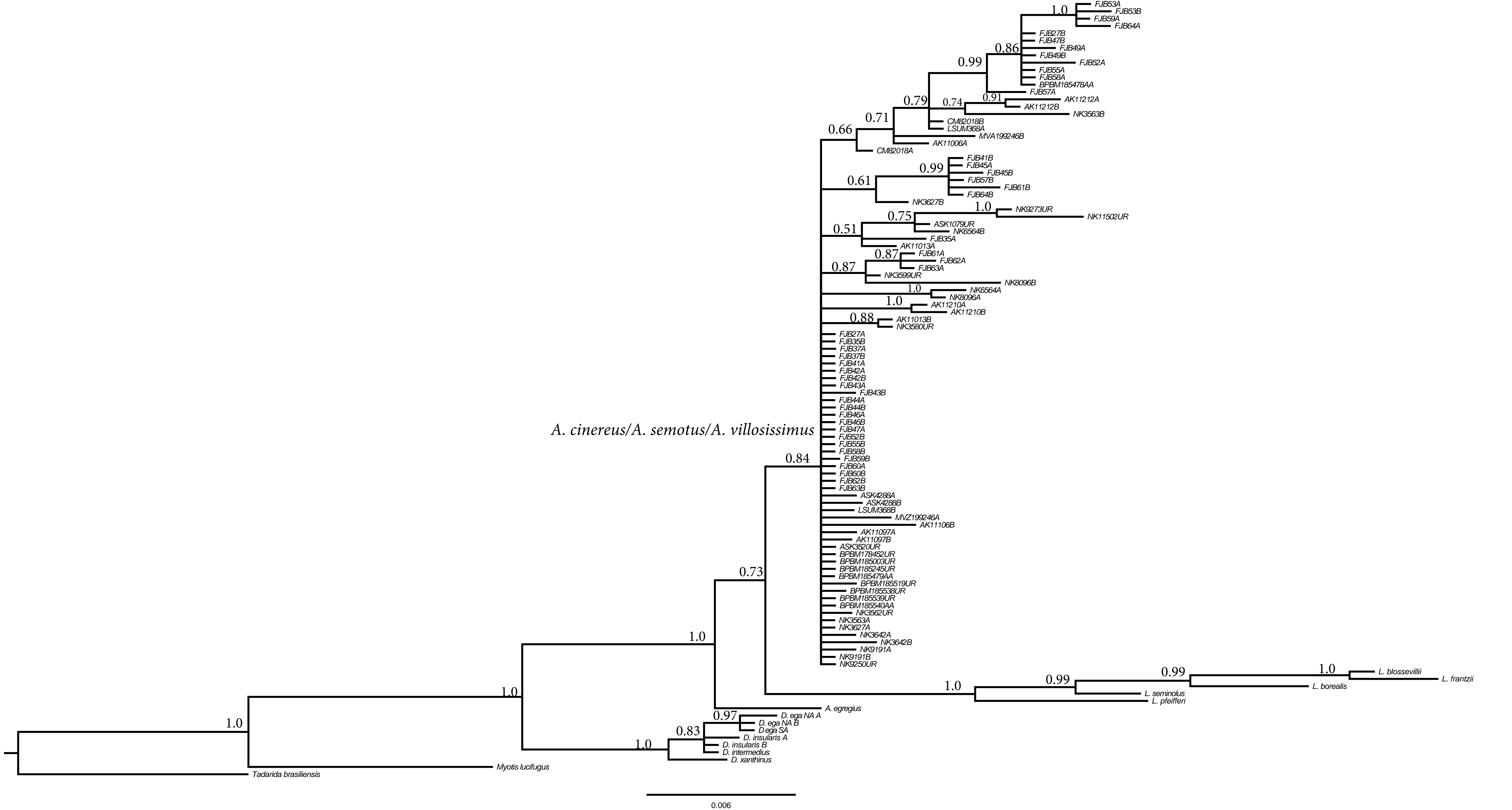

Supplement: S6 Fig — Where available, individual alleles were used in the analysis. Numbers at nodes represent Bayesian posterior probabilities. Letters following sample names indicate alleles: “A” and “B” represent two different alleles from the same specimen; “AA” represents a homozygous specimen; “UR” represents unresolved alleles (in this case, the consensus sequence of the two alleles was used in the analysis). (PDF) [file pone.0186085.s006.pdf]
